# Effect of Supplementary Light with Different Wavelengths on Anthocyanin Composition, Sugar Accumulation and Volatile Compound Profiles of Grapes

**DOI:** 10.3390/foods12224165

**Published:** 2023-11-17

**Authors:** Junxia Zhang, Wanping Li, Peng Zhang, Xuehao Zhang, Jinfeng Wang, Lujun Wang, Keqin Chen, Yulin Fang, Kekun Zhang

**Affiliations:** 1 Heyang Viti-Viniculture Station, Ningxia Helan Mountain’s East Foothill Wine Experiment and Demonstration Station, College of Enology, Northwest A&F University, Yangling 712100, China; junxia@nwafu.edu.cn (J.Z.); liwanping@nwafu.edu.cn (W.L.); 13032920527@163.com (P.Z.); zhangxuehao@nwafu.edu.cn (X.Z.); chenkeqin1985@nwafu.edu.cn (K.C.); fangyulin@nwsuaf.edu.cn (Y.F.); 2 Weinan Grape Research Institute, Weinan 714000, China; 18591397059@163.com (J.W.); wnwlj@163.com (L.W.)

**Keywords:** supplemental lighting, grape, anthocyanin, sugar, aroma

## Abstract

Protected cultivation is currently one of the main cultivation modes for grape production, but the long-term use of plastic film will have a certain negative impact on the light environment in vineyards, which in turn causes poor colouring, low sugar content and a lack of aroma in some red grape varieties. Supplementing light can be an effective way to mitigate these problems. In this study, vines of three red table grape varieties (‘Summer Black’, ‘Xinyu’ and ‘Queen Nina’) cultivated in a plastic greenhouse were supplemented with red, blue, white and red-blue light from veraison to harvest. All four supplemental light treatments increased the content of anthocyanins, sugars and volatile compounds in three grape varieties compared to CK (no supplemental lighting). Red-blue light treatment was the most favourable for the accumulation of anthocyanins and sugars, and the grapes treated with blue light had the highest content of volatile compounds. The grapes treated with red-blue light all obtained the highest composite scores via principal component analysis. For most of the sensory properties, the highest scores were obtained by the red-blue light-treated grapes. The results of this study will be useful in improving the colouring, sugar, and aroma content of grapes under protected cultivation.

## 1. Introduction

Grapes are one of the world’s most popular fruit species, with about 76,000 square kilometres of agricultural land devoted to their production globally, of which about 21 percent is table grapes [1,2]. However, in some areas of high precipitation, such as southern China, excessive rainfall causes grape berries to be more susceptible to disease, which in turn affects the commercial value of fruits [3]. Rain shelters, plastic tunnels, solar greenhouses, and other protected cultivation modes have been widely adopted by producers, and these cultivation modes are not only conducive to the prevention of natural disasters and the control of pests and diseases, but also expand the cultivation area of good varieties, prolong the supply period of fresh fruits, and improve the economic benefits of farmers [4,5,6]. However, the long-term use of plastic films can reduce the light intensity in vineyards and change the light quality environment, which in turn affects the quality of grapes [7].

Light is the energy source for plant photosynthesis and has a significant effect on plant morphogenesis, physiological metabolism, and gene regulation [8]. Changes in light conditions can affect the concentration and composition of anthocyanins in grape skins [9], the accumulation of carbohydrates in fruits [10], and the biosynthesis and accumulation of aroma substances by regulating the expression of key genes in the synthesis pathway of some volatile compounds [11]. Therefore, improving the light environment in the facility is of great significance to grape production.

In recent years, several cultivation management measures have been employed to regulate light conditions in the fruiting zone, such as vineyard trellis management, pruning [12], changes in row orientation [13], leaf removal [14], and bagging [15]. However, some of these measures have limited effect on improving light conditions, and some carry a potential risk of sunburning the grapes. Some studies have found that exposing plants to different wavelengths of light-emitting diode (LED) can effectively promote fruit ripening [16], increase the nutrient content of fruit [17,18,19], and improve fruit quality [20]. Red-blue light promoted the accumulation of total carbohydrates, starch, and sucrose in tomatoes [21]; supplementation of greenhouse-grown peas with red-blue light promoted their growth and increased the content of soluble sugars [22]; and in experiments on sweet oranges, ultraviolet light and red-blue light accelerated fruit ripening and affected the content of organic acids, hexoses, and carotenoids in fruit [23]. However, these studies have been scattered across different horticultural crops, and few trials have been conducted to systematically study table grapes [24,25]. In addition, most of the previous studies were conducted with a single species as the test material, leading to a lack of credibility of the results.

Therefore, facility-cultivated fuchsia table grapes ‘Summer Black’, ‘Xinyu’, and pink table grape ‘Queen Nina’ were used as experimental materials in this study to investigate the effects of supplemental red (RE), blue (BLU), white (WH), and red-blue (R-B) light on the quality of grapes in terms of anthocyanins, sugars, and volatile compounds, which is of great significance for the improvement of the environment of the facility and for improving the quality of grape berries.

## 2. Materials and Methods

### 2.1. Vineyard Site, Materials and Experimental Layout

This study was conducted at the Weinan Grape Research Institute (Weinan, Shaanxi, China, 35°52′ N, 108°50′ E) in the connective plastic greenhouse during the 2022 growing season. The vineyard is located at an altitude of 349 m above sea level and has a warm-temperate semi-arid monsoon climate, with an average annual temperature of 13.5 °C, an average annual precipitation of about 550 mm, an average annual sunshine of 2277 h, and a frost-free period of 199–255 d. The soil type of the vineyard is sandy loam.

The three table grapes used in this study were 8-year-old facility grown Summer Black (SB), Xinyu (XI), and Queen Nina (QN), all red grape varieties, with ‘T’ type trellis, north-south rows, 1.5 m × 3.0 m spacing. The pruning method was a single bud per spur. The four wavelength LEDs used in this study were red light (RE), blue light (BLU), white light (WH), and red-blue light (R-B), made by Shanghai Heming Lighting Co. with the model number of T8-18W. The wavelengths of the RE and BLU were 650 nm and 450 nm, respectively. The WH was a compound light with multiple wavelengths. The R-B was a combination light, which was made by combining the number of red LEDs and blue LEDs in a 1:1 ratio.

The LEDs were mounted above the canopy of the vines, with two lamps above each cane, and the position of the lamps was adjusted so that the light intensity around the clusters of each treatment was 3000 Lx at night. In the vineyard, three vines per treatment group were used as replicates. In order to avoid the interaction of lamps of different wavelengths, vines at a sufficient distance were used for the experiment; this distance was determined by measuring the light intensity at night using an illuminance meter (HP-350, Hangzhou, China). The experiment began with supplemental light treatments on vines at E-L 34 (when berries began to soften and sugar levels began to increase) and continued until the end of the harvest period, with daily irradiation times of 3:00~7:00 and 19:00~23:00. This experiment was set up with treatment groups supplemented with RE, BLU, WH, and R-B, as well as a CK (no supplemental lighting) group.

### 2.2. Sample Collection

Approximately 100 berries were randomly collected (top, middle, and bottom of the clusters) from each treatment group, and half of them were from different orientations. Immediately thereafter, the samples were kept in an insulated cooler and transported to the laboratory within one hour to be snap-frozen in liquid nitrogen and stored in a −80 °C refrigerator for subsequent assays. Sampling times were referenced to the E-L system [26]. A total of 5 samples were collected from E-L 34 to E-L 38. The stages are: berries begin to soften and Brix starts increasing (E-L 34), berries begin to colour and enlarge (E-L 35), berries with intermediate Brix values (E-L 36), berries not quite ripe (E-L 37), berries harvest-ripe (E-L 38).

### 2.3. Monitoring of Light Intensity

A sunny and cloudless day was chosen to determine the light intensity inside and outside the greenhouse and around the clusters in the canopy. An illuminance meter (HP-350, Hangzhou, China) was used for the measurements, which started at 3:00 and ended at 23:00, and the data were recorded at 30 min intervals.

### 2.4. Monitoring of Fruit Ripening

The ripening of grapes was monitored by determining the soluble solids and pH of the fruit. About 20 grapes stored in a refrigerator at −80 °C were randomly selected, crushed, and filtered through gauze to obtain the juice. Soluble solids and pH were determined using a pocket saccharimeter (PAL-1, Atago, Tokyo, Japan) and a pH meter (FE28, Mettler Toledo, Shanghai, China), respectively, and three biological replicates were carried out for each treatment group.

### 2.5. HPLC-MS Analysis of Individual Anthocyanins in Grapes

The extraction and determination of individual anthocyanins were carried out with reference to the previous methods [27]. Berries (a total of 50) were randomly selected from each treatment group and manually peeled. The peel was ground with liquid nitrogen and freeze-dried in a vacuum freeze-dryer (FD5 series, GOLD-SIM, Seattle, WA, USA). 0.50 g of lyophilised dried grape skin powder was weighed and extracted with 10 mL of hydrochloric acid/water/methanol solution (1:19:80, *v*/*v*/*v*). The extraction was carried out sequentially using an ultrasonic machine, a shaker, and a centrifuge under light-free conditions, and then the supernatant containing individual anthocyanins was collected. The whole process was repeated four times, and the supernatant (about 40 mL) collected from the four times was evaporated to dryness using a vacuum concentrator, then fixed to 5 mL with chromatographic methanol, and finally filtered through a 0.45 µm nylon filter membrane into the injection vial and then assayed on the machine.

Liquid chromatographic analysis was performed using high-performance liquid chromatography (HPLC, LC-20 A, Shimadzu, Kyoto, Japan), and the samples were separated on a C-18 column (250 × 4.6 mm, 4 μm, SynergiTM 4 µm Hydro-RP 80 A, Phenomenex, Torrance, CA, USA). The composition of mobile phase A: water/acetonitrile/formic acid (32:4:1, *v*/*v*/*v*); mobile phase B composition: water/acetonitrile/formic acid (16:20:1, *v*/*v*/*v*). The solvent gradient was eluted as follows: 0.00–15.00 min, 0–10% B; 15.00–30.00 min, 10–20% B; 30.00–45.00 min, 20–35% B; 45.00–46.00 min, 35–100% B; and 50.00–51.00 min, 100–0% B. The elution was carried out with the mallow equivalents of anemone-3-*O*-glucoside to express the relative concentration of each individual anthocyanin.

### 2.6. HPLC Analysis of Hexose Contents in Grapes

Hexose (glucose and fructose) content was determined by HPLC. The juice was extracted from randomly selected grape berries from each treatment by squeezing and filtering with four layers of gauze, then diluted 50-fold with ultrapure water, and then filtered through a 0.22 μm organic filter membrane into a glass injection bottle for on-line testing.

Column: ZORBAX Carbohydrate (4.6 mm × 250 mm, 5 μm); Mobile phase: acetonitrile:water = 78:22 (*v*/*v*); Flow rate: 1.0 mL/min; Drift tube temperature: 100 °C; injection volume: 20 μL; column temperature: 30 °C; detector: Differential refractive index (DIRI); Analysis time: 35 min. All chemicals and standards were purchased from Sigma (St. Louis, MO, USA) and dissolved in deionised water. The standards used in this work include D- (+) -glucose and D- (−) fructose. Three technical replicates were analysed per sample.

### 2.7. GC-MS Analyses of Volatile Compounds in Grapes

The extraction of volatile substances from grape berries was carried out with reference to the previous method [7]. Approximately 25 grapes stored in a −80 °C refrigerator were randomly selected for each treatment group, and the grapes were crushed under the protection of liquid nitrogen to remove seeds. Then 0.5 g of D-gluconolactone and 1 g of polyvinylpyrrolidone were added to the crushed grapes and quickly powdered with a grinder (A11BS025, IKA, Königswinter, Germany), then loaded into 50 mL centrifuge tubes and macerated overnight in the refrigerator at 4 °C to allow sufficient extraction of volatile compounds. After centrifugation the next day, 5 mL of supernatant, 1 g of NaCl, and 10 µL of internal standard (4-methyl-2-pentanol) were added to the glass vials for the on-board assay.

An Agilent gas chromatography (GC) model 7890 instrument equipped with an Agilent 5975 mass spectrometry (MS), a 7683 automatic sampler (Agilent, Santa Clara, CA, USA), and a DB-Wax capillary column (60 m × 0.25 mm × 0.25 µm; Agilent J & W, Santa Clara, CA, USA) was used for the analysis. The temperature increase procedure was as follows: the initial temperature was 50 °C, then held for 5 min, then increased to 220 °C at a rate of 3 °C/min, and finally held for 5 min, with an analysis time of 70 min. Three technical replicates were analysed per sample.

### 2.8. Sensory Description Analysis of Grapes

Sensory analysis of experimental grapes was carried out in a standard sensory analysis chamber (College of Enology, Northwest A&F University, China) according to previously established approaches with some modifications [28]. The panel was made up of 14 experienced tasters, aged between 21 and 35 (7 women and 7 men), with specialised sensory abilities to identify and characterise grapes. Panellists scored the grapes using a 5-point scoring system according to the following descriptors: A, appearance (colour intensity); B, taste (sweetness, acidity, taste balance); C, aroma (aroma intensity); and D, texture (skin edibility, fruit texture). The grapes were analysed in triplicate.

### 2.9. Statistical Analysis

Statistical analysis was performed with the software SPSS Statistics 22.0 (IBM, Armonk, NY, USA). Significant differences among the treatments at the same developmental stage were analyzed by a one-way ANOVA followed by the Duncan’s multiple comparison test at *p* < 0.05. All errors are expressed as the standard deviation (SD) of triplicate analyses for each treatment group, and the values are presented as the mean ± SD (*n* = 3). The normalized raw data of 18 metabolites after taking the Z-score were used for correlation analysis and principal component analysis (PCA) [29]. PCA was analyzed using a correlation matrix and between-groups algorithm method. Figures were constructed via Origin 2021 (OriginLab Corporation, Northampton, MA, USA) and GraphPad Prism 8 (GraphPad Software, 225 Franklin Street. Fl. 26, Boston, MA, USA).

## 3. Results

### 3.1. Variation of the Light Intensity during a Day

In order to clarify the effect of the plastic film used in this study on the ambient light intensity inside the greenhouse and the effect of supplemental light on the light intensity inside the canopy, the light intensity inside and outside the greenhouse and inside the canopy before and after supplemental light was monitored for 20 h (Figure S1), which was conducted on a clear and cloudless day after the experiments were set up. It was found that the light intensity inside the greenhouse was significantly reduced by the presence of the plastic film and that the light intensity inside the canopy was much lower than the ambient light intensity. Therefore, supplemental light to the vines in this greenhouse is necessary. The light intensity inside the canopy increased significantly after nighttime supplemental lighting of the vines using LEDs.

### 3.2. Effects of Supplemental Lighting with Different Wavelengths on Grape Ripening

**Figure 1:**
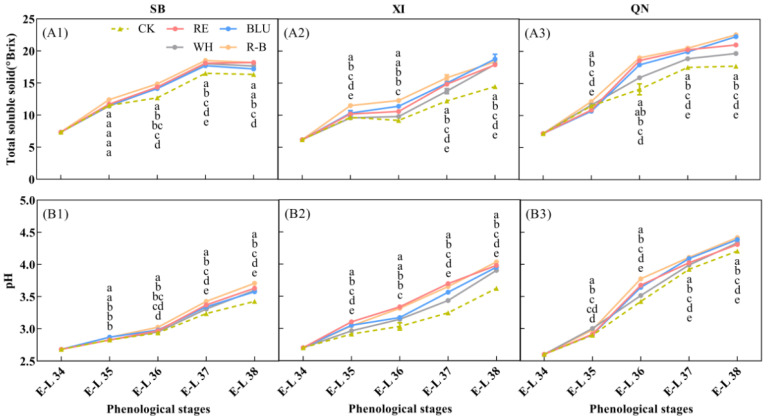
Total soluble solid (**A1**–**A3**) and pH (**B1**–**B3**) in grapes of different treatments across 5 phenological stages. CK: no light treatment; RE: red light treatment; BLU: blue light treatment; WH: white light treatment; R-B: red and blue light treatment (1:1). The error bars show the standard deviation of the mean (*n* = 3). Different letters indicate significant differences among treatments by one-way ANOVA (*p* < 0.05).

Changes in soluble solids content and pH of grape berries from different treatments were determined (Figure 1). It can be seen that the soluble solids content accumulated as the fruit development process progressed (Figure 1A). At the initial stage of supplementing the vines with light (E-L 34), the fruits had a low soluble solids content of about 6–8 °Brix, which did not differ between treatments. After entering the veraison stage, significant differences in soluble solid content were observed between treatments in XI and QN, while the differences between treatments in SB remained non-significant, most likely because of the faster developmental process of the SB berries, so that supplemental light did not have enough time to have a significant effect on soluble solids content by the time the berries entered the veraison stage. The differences in soluble solids content between treatments for each variety gradually increased when entering E-L 36. By the time of harvest (E-L 38), soluble solids content peaked in all treatments for XI and QN and decreased slightly for SB. Throughout the colour change stage (from E-L 35 to E-L 37), the R-B treatment had the highest berry soluble solids content of all three grape varieties, and the CK had the lowest berry soluble solids content. Specifically, from E-L 35 to E-L 37, soluble solids content of SB, XI and QN berries increased by 49.19%, 37.68% and 68.03% in R-B treatment, respectively. Correspondingly, CK increased by 43.47%, 26.56%, and 52.17%, respectively. At the harvest stage, soluble solids content of R-B treated SB, XI, and QN berries increased by 11.43%, 26.71%, and 27.68%, respectively, compared to CK. The pH of the berries of the three varieties increased as the grapes developed (Figure 1B). For SB, the pH of the berries was low, from E-L 34 to E-L 36, and the difference between treatments was small. When entering E-L 36, the pH of the berries increased rapidly, and the difference between treatments gradually increased. Berry pH of XI and QN increased progressively throughout the developmental stages of the grapes and varied significantly among treatments. By the time of the harvest stage (E-L 38), berry pH peaked in all treatments for all three varieties. Overall, berry pH was higher in the R-B treatment than the other treatments at the harvest stage, with the RE, BLU, and WH treatments having intermediate pH values and CK having the lowest pH value.

### 3.3. Individual Anthocyanins and Total Anthocyanin Content (TAC) in Grapes during Ripening

**Figure 2:**
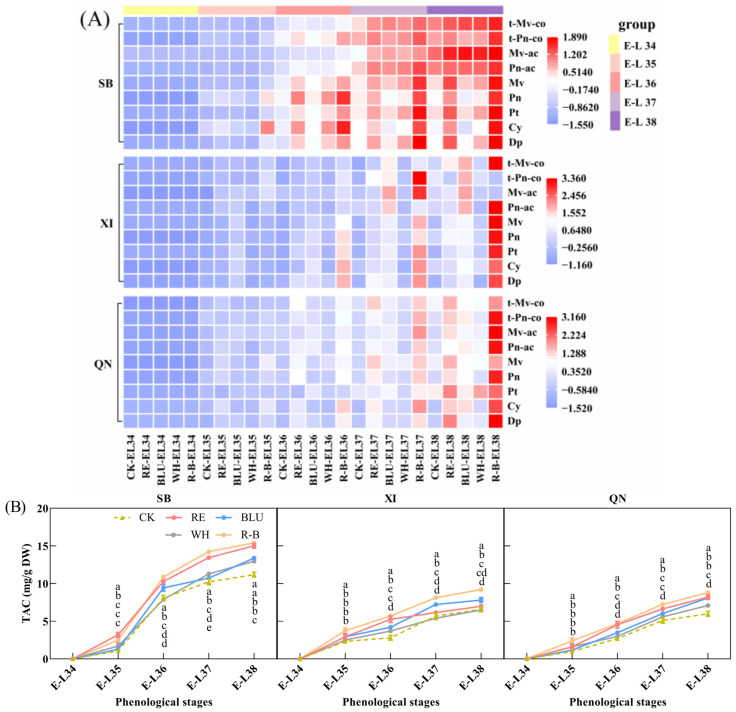
(**A**) Heat map of the content of individual anthocyanins in grapes from different treatments at five phenological stages. The data were Z-score normalised using Origin software 2021. Different cell colours in the heatmap represent a high concentration (red) or a low concentration (blue). Abbreviations: CK, no light treatment; RE, red light treatment; BLU, blue light treatment; WH, white light treatment; R-B, red and blue light treatment (1:1); Dp, delphinidin-3-*O*-glucoside; Cy, cyanidin-3-*O*-glucoside; Pt, petunidin-3-*O*-glucoside; Pn, peonidin-3-*O*-glucoside; Mv, malvidin-3-*O*-glucoside; Pn-ac, peonidin-3-*O*-(6-*O*-acetyl)-glucoside; Mv-ac, malvidin-3-*O*-(6-*O*-acetyl)-glucoside; t-Pn-co, trans-peonidin-3-*O*-(6-*O*-p-coumaryl)-glucoside; t-Mv-co, trans-malvidin-3-*O*-(6-*O*-p-coumaryl)-glucoside. (**B**) Total anthocyanin content of grapes from different treatments across 5 phenological stages. Each anthocyanin concentration was calculated on the basis of dry weight of the grape skin. Error bars are the standard deviation of the mean (*n* = 3). Different letters indicate significant differences among treatments using Duncan’s test (*p* < 0.05). Abbreviations: TAC, total anthocyanin content; DW, skin dry weight.

Anthocyanins are water-soluble substances that determine the colour of grapes [30,31]. In this study, nine major individual anthocyanin contents (Figures S2–S4) and total anthocyanin content (Figure 2B) were determined in the pericarp of developing SB, XI, and QN grape berries, and changes in individual anthocyanin content during development were plotted as a heat map (Figure 2A). The results showed that the contents of nine individual anthocyanins and total anthocyanin in the skins of the three grape varieties accumulated as fruit development progressed and reached a peak at fruit ripening. In addition, there were differences in total anthocyanin levels among the varieties at the maturity stage, with SB having a higher total anthocyanin content than SB and XI, and XI and QN having similar total anthocyanin levels. In addition, there were differences in total anthocyanin levels among the varieties at the maturity stage, with SB having higher total anthocyanin levels than XI and QN, which were close to each other. For SB, the individual anthocyanin content and total anthocyanin content were lower for each treatment from E-L 34 to E-L 35, and the differences between treatments were small. Upon entering veraison, the content of individual anthocyanin and total anthocyanin increased rapidly and showed significant differences among the treatments up to the harvesting stage. Among them, the total anthocyanin content of SB fruits from R-B treatment was the highest, followed by RE treatment. In addition, in terms of individual anthocyanin content, R-B supplementation increased the content of five basic individual anthocyanins (including Dp, Cy, PT, Pn, and Mv), two acetylated individual anthocyanins (Pn-ac and Mv-ac), and two coumaroylated individual anthocyanins (t-Pn-co and t-Mv-co). RE supplementation increased the content of five glycosylated anthocyanins and one acetylated individual anthocyanin (Mv-ac).

For XI, the rate of total anthocyanin accumulation was more stable during fruit development. From the E-L 35 stage onwards, the R-B treatment was observed to be favourable for total anthocyanin accumulation, forming a significant difference from the other treatments. Thereafter, the differences between treatments gradually increased, with the R-B treatment consistently having the highest anthocyanin content in XI grape clusters until harvest. In addition, in terms of the content of individual anthocyanins, all nine individual anthocyanin contents were higher in the R-B supplemental light treatment than in the other treatments at harvest.

For QN, the trend of total anthocyanin content during development was similar to that of XI. During the E-L 35 period, the total anthocyanin content of QN grape berries from the R-B treatment was higher than that of the other treatments, whereas the differences in total anthocyanin content among the other treatments were not significant. After entering E-L 36, the differences in total anthocyanin content between treatments gradually increased, with the R-B treatment remaining the most favourable for total anthocyanin accumulation, followed by the RE treatment, and this continued until harvest. In addition, in terms of the content of individual anthocyanins, both the R-B and RE treatments increased the content of these nine individual anthocyanins at harvest time, with the R-B light supplementation treatment having a higher content of nine individual anthocyanins than the other treatments, and the BLU treatment increasing the content of four basic individual anthocyanins (Dp, Cy, Pn, and Mv), two acetylated individual anthocyanins, and two coumaroylated individual anthocyanins.

Overall, the R-B treatment was most favourable for the accumulation of the nine major individual anthocyanins and total anthocyanin in the skins of SB, XI, and QN grapes, followed by the RE treatment.

### 3.4. Hexose Content of Grapes during Ripening

**Figure 3:**
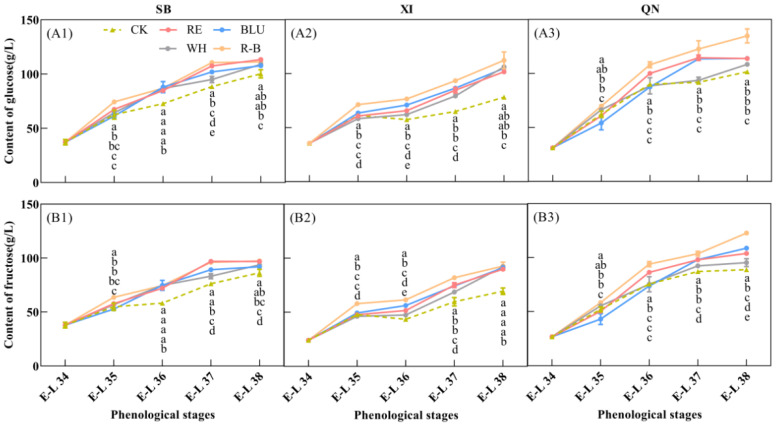
Glucose (**A1**–**A3**) and fructose (**B1**–**B3**) content of grapes from different treatments at 5 phenological stages. Each hexose content was calculated on the basis of fresh tissues of the whole berries. Error bars are the standard deviation of the mean (*n* = 3). Different letters indicate significant differences among treatments using Duncan’s test (*p* < 0.05). Abbreviations: CK, no light treatment; RE, red light treatment; BLU, blue light treatment; WH, white light treatment; R-B, red and blue light treatment (1:1).

Sugar fractions are important indicators for evaluating the quality of grape berries, of which glucose and fructose are the two most predominant sugars in ripe grape berries, and both are hexoses with a content of about 1:1 [32]. In this study, glucose (Figure 3A) and fructose contents (Figure 3B) were determined in developing SB, XI, and QN fruits. The results showed that the glucose and fructose contents in the fruits of the three varieties used in the experiment accumulated as fruit development progressed and peaked at fruit maturity. In addition, there were differences in hexose levels among the varieties at the ripening stage. Glucose and fructose levels were higher in QN than in SB and XI, and glucose and fructose levels were close to each other in SB and XI, which was in keeping with the differences in soluble solids content among the three varieties at the ripening stage (Figure 1A).

For SB, glucose and fructose accumulated rapidly in berries from E-L 34 to E-L 37, and the rate of glucose and fructose accumulation slowed down after entering E-L 37 until harvest. In addition, there were differences in the hexose content of SB berries treated with different wavelengths of light supplementation at harvest time. The glucose content of SB berries was higher in RE, BLU, and R-B treatments at 113.34 g/L, 107.63 g/L, and 111.22 g/L, respectively. RE and R-B treated SB berries had a higher fructose content of 96.75 g/L and 97.27 g/L, respectively.

For XI, the fastest rate of hexose accumulation was observed from E-L 34 to E-L 35, which became slower after entering veraison, and then started to accumulate rapidly again after entering E-L 36 until harvest. In addition, there were differences in the hexose content of XI grape berries treated with different wavelengths of light supplementation at harvest time. The highest glucose and fructose contents were found in XI berries treated with R-B at 112.33 g/L and 92.7 g/L, respectively.

For QN, the hexose content showed a tendency to increase rapidly at the beginning and then slow down with the developmental period of the fruit, and E-L 36 was the cut-off point at which the rate of hexose accumulation changed. In addition, there were differences in the hexose content of QN grape berries treated with different wavelengths of light supplementation at harvest. The highest glucose and fructose content of QN berries in the R-B treatment was 134.81 g/L and 122.67 g/L, respectively, which formed a significant difference between the other treatments.

Overall, the R-B treatment was most favourable for glucose and fructose accumulation in SB, XI, and QN grape berries.

### 3.5. Volatile Compounds in Grapes at Harvest

Volatile compounds are important flavour substances in grape berries and are mainly found in the skins [33]. Table 1 shows the identified and quantified results of free volatile compounds in grapes at harvest. A total of 53 volatile compounds were detected in SB, mainly including esters, terpenes, high alcohols and aldehydes, and ketones. Among them, the high alcohols have the largest variety of volatile compounds, which are 21 in number, accounting for 25.29–35.47% of the total volatile compounds. The esters had the highest content of volatile compounds, accounting for 34.17–50.35% of the total volatile compounds. There were differences in the volatile compound content of SB grape berries treated with different wavelengths of light supplementation at the harvest stage. Among them, the content of volatile compounds such as esters, high alcohols and aldehydes, and ketones was significantly higher in BLU-treated SB berries than in the other treatments. Esters confer abundant floral and tropical fruit aromas to grapes. Among esters, ethyl acetate was the most abundant compound and generated an ethereal, fruity aroma [34]. The terpene volatile compounds content of R-B-treated berries was significantly higher than the other treatments, especially α-terpineol, geraniol, and (*Z*)-3,7-dimethyl-2,6-octadien-1-ol.

A total of 42 volatile compounds were detected in XI berries, which, like those in SB berries, can be classified into four main groups: esters, terpenes, high alcohols and aldehydes, and ketones. Among them, the high alcohols had the largest variety and highest content of volatile compounds, with 18 types, accounting for 48.92–58.62% of the total volatile compounds. There were differences in the volatile compound content of XI berries treated with light supplementation at different wavelengths at harvest. Among them, the content of volatile compounds of esters and high alcohols of XI berries was significantly higher in BLU treatment. The content of terpene volatile compounds was significantly higher in XI berries from the R-B treatment than from the other treatments, especially γ-terpinene. The content of aldehydes and ketones as volatile compounds was significantly higher in RE-treated XI berries than in other treatments, especially 2-hexenal.

A total of 45 volatile compounds were detected in the QN berries, which were classified into four main groups—esters, terpenes, high alcohols and aldehydes, and ketones—as in the other two varieties. Among them, the high alcohols had the largest variety and highest content of volatile compounds, with 19 types, accounting for 36.84–42.12% of the total volatile compounds. At the harvest stage, there were differences in the volatile compound content of QN grape berries treated with different wavelengths of light supplementation. Among them, BLU-treated QN berries had a significantly higher content of esters, high alcohols and aldehydes, and ketones volatile compounds than the other treatments.

Overall, BLU treatments were most favourable for the accumulation of volatile compounds in SB, XI, and QN grape berries.

### 3.6. Correlation Analysis of Fruit Quality Indexes

**Figure 4:**
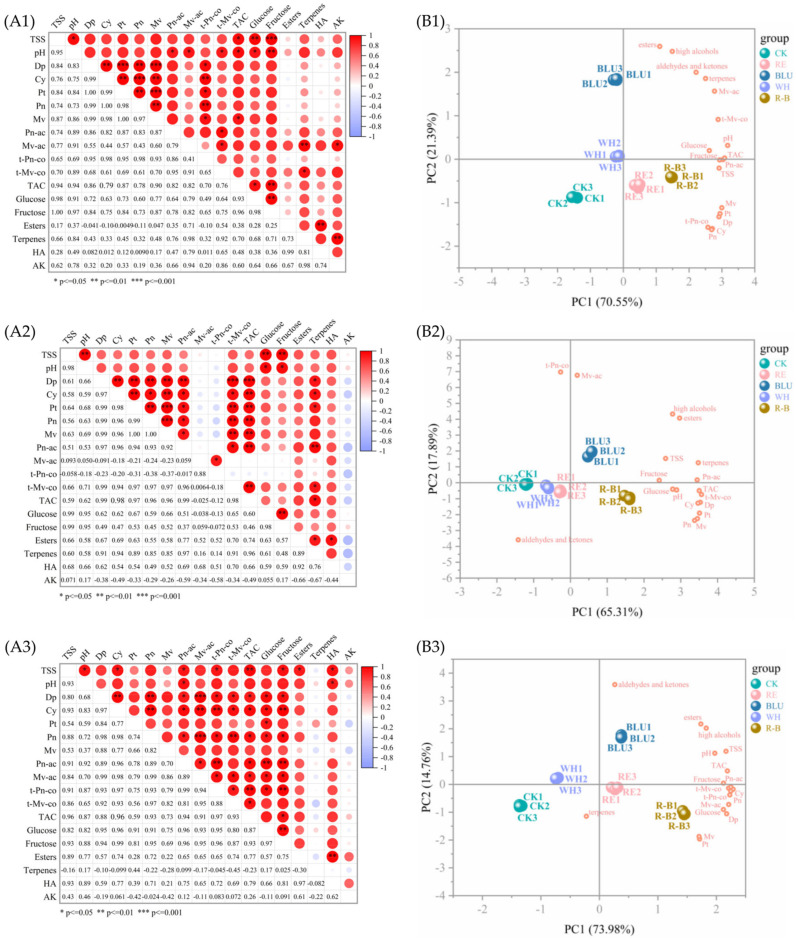
(**A1**–**A3**) Correlation analysis of 18 quality indicators in SB, XI, and QN. The legend on the right represents the correlation between indicators from low (deep red) to high (deep blue), and the numbers in the bottom left of the heatmap represent the percentage of correlation coefficients. Abbreviations: CK, no light treatment; RE, red light treatment; BLU, blue light treatment; WH, white light treatment; R-B, red and blue light treatment (1:1). (**B1**–**B3**) Loading values of the grape quality indicators in the two PCs and the distribution of different treatments in SB, XI and QN.

Correlation analyses of the 18 quality indicators of SB, XI, and QN grape berries, respectively, revealed that each indicator reflected the quality of the grape berries to a different extent while at the same time being correlated with each other to a certain extent. The Pearson correlation coefficient was used to analyse the correlation between these 18 indicators, and the results are shown in Figure 4A.

For SB, there is basically a positive correlation between all 18 indicators. Among them, there was a highly significant positive correlation (*p* ≦ 0.01 or even *p* ≦ 0.001) between some of the individual anthocyanins; between total soluble solid (TSS) and hexose, between pH and fructose, between TAC and fructose, between glucose and fructose, between esters and high alcohols, between terpenes and Mv-ac, and between terpenes and aldehydes and ketones were all highly significant positive correlations (*p* ≦ 0.01).

For XI, there was no significant negative correlation between the 18 indicators. Among them, highly significant positive correlations (*p* ≦ 0.001) were found between some of the individual anthocyanins and between total anthocyanin and Dp. There were highly significant positive correlations (*p* ≦ 0.01) between TSS and pH, between TSS and hexose, between glucose and fructose, and between terpenes and Pn-ac.

For QN, there was no significant negative correlation between the 18 indicators. Among them, there was a highly significant positive correlation (*p* ≦ 0.01 or even *p* ≦ 0.001) between some of the individual anthocyanins. There were highly significant positive correlations (*p* ≦ 0.01) between TSS and TAC, between Pn-ac and hexose, between Cy and fructose, between t-Pn-co and fructose, and between glucose and fructose.

Overall, there were no significant negative correlations between the 18 quality indicators of SB, XI, and QN grape berries.

### 3.7. Principal Component Analysis

From Figure 4A, it can be seen that there are obvious correlations among the quality indicators of SB, XI, and QN grape berries, and if the related indicators are directly used to evaluate the quality of the berries, a large amount of information overlap will occur, while the principal component analysis is able to downscale a variety of variables with strong correlations, convert the original variables into new effective principal components, and retain the total variation of the data as much as possible in a small number of principal components [35]. Therefore, 18 quality indicators related to grape fruit sugar fractions, anthocyanins, and aroma were selected for principal component analysis in this study (Figure 4). The eigenvalues and variance contributions of the 18 principal components of these 3 varieties are shown in Table S1. Taking eigenvalues ≧1.00 as the standard according to the well-known Kaiser’s rule, the cumulative variance contributions of the first 2 principal components of SB and the first 3 principal components of XI and QN were 91.936%, 97.276%, and 97.979%, respectively, which basically reflected the information contained in the raw data. Therefore, the 18 quality indicators of SB, XI, and QN were simplified into two, three, and three principal components, respectively, and then the comprehensive quality evaluation was carried out with the help of these principal components. As can be seen from Figure 4 and Figure S5, the three biological replicates of each wavelength of light treatment were clustered together, while the samples of different groups were more discrete, which indicates that different wavelengths of light treatments had a greater effect on the quality parameters of grapes.

The degree of influence of the principal components can be expressed by the variables in the principal component loading matrix [36], so the data are rotated so that the factors tend to be bifurcated, which is more conducive to further determining the actual meaning of the factors. The loading matrices for the first 2 principal components of SB and the first 3 principal components of XI and QN are shown in Table S2.

From Tables S1 and S2, it can be seen that among the 2 principal components of SB, the eigenvalue of PC1 is 12.699 and the variance contribution rate is 70.551%, which indicates that PC1 plays a dominant role in the analysis and evaluation. Among them, pH, TAC, fructose, and Pn-ac had higher loading values, and all four indicators positively affected PC1. Thus, PC1 mainly reflects the sugar, acid, and anthocyanin levels of SB. As can be seen from Figure 4B1, the esters, high alcohols, aldehydes and ketones, and terpenes parameters are clustered together and located in the first quadrant, indicating that they are strongly correlated, which is consistent with the findings obtained in the correlation analysis. In addition, the Mv, Pt, Cy, Dp, Pn, and t-Pn-co clustered together and were located in quadrant 4, indicating that they are strongly correlated, which is also consistent with the findings obtained in the correlation analysis. R-B and RE-treated samples were in the positive direction of the PC1 axis, suggesting that they had a greater tendency to increase the sugar, acid, and anthocyanin levels than BLU, WH, and CK. The eigenvalue of PC2 was 3.849 with a variance contribution of 21.385%, where esters, high alcohols, aldehydes and ketones, and terpenes positively loaded weights were larger, thus PC2 reflects the aroma of SB. BLU-treated samples were in the positive direction of the PC2 axis, which indicates that it tends to improve the content of aroma more than other treatments.

Among the 3 principal components of XI, PC1 has an eigenvalue of 11.755 and a variance contribution rate of 65.305%, indicating that PC1 plays a dominant role in the analysis and evaluation. Among them, t-Mv-co, TAC, Dp, and Pt had higher loading values, and all four indicators positively affected PC1. Thus, PC1 mainly reflects the individual anthocyanin of XI. As can be seen from Figure 4B2, the grouping of variables was similar to that in SB; aroma-related parameters were clustered together and located in the first quadrant, and anthocyanin-related parameters were clustered together and located in the fourth quadrant. In addition, sugar- and acid-related metrics were clustered in the positive semi-axis of PC1, indicating a strong correlation between them. R-B and BLU-treated samples were located in the positive direction of the PC1 axis, suggesting a tendency for R-B and BLU to be more able to increase the content of individual anthocyanins compared to the other treatments. The eigenvalue of PC2 was 3.221 with a variance contribution of 17.892%, where t-Pn-co, Mv-ac, high alcohols, and esters positively loaded with higher weights, thus PC2 reflects the individual anthocyanin and aroma of XI. As can be seen from Figure 4B2, BLU-treated samples were in the positive direction of the PC2 axis, which indicates that it tends to improve individual anthocyanin and aroma more than other treatments. The eigenvalue of PC3 was 2.534 with a variance contribution of 14.079%, where fructose, Tss, pH, glucose and aldehydes, and ketones positively loaded with higher weights, thus PC3 reflects the sugar fractions and aroma of XI. As can be seen from Figure S5B4, the sugar- and acid-related parameters are well clustered in the first quadrant, indicating a strong correlation between them. RE and WH-treated samples were in the positive direction of the PC3 axis, suggesting that they had a greater tendency to increase these parameters than BLU, R-B, and CK.

Among the 3 principal components of QN, PC1 has an eigenvalue of 13.316 and a variance contribution rate of 73.980%, indicating that PC1 plays a dominant role in the analysis and evaluation. Among them, Cy, fructose, t-Pn-co, and Pn had higher loading values, and all four indicators positively affected PC1. Thus, PC1 mainly reflects the individual anthocyanin and sugar fractions of QN. As can be seen from Figure 4B3, sugar- and acid-related parameters are dispersed in the positive semiaxis of PC1, and PC2 separates aroma- and anthocyanin-related parameters well, with aroma-related parameters clustered in the first quadrant and anthocyanin-related parameters clustered in the fourth quadrant. BLU, R-B, and RE-treated samples were in the positive direction of the PC1 axis, suggesting that they had a greater tendency to increase the individual anthocyanin and sugar fractions of QN than CK and WH. The eigenvalue of PC2 was 2.656 with a variance contribution of 14.756%, where the weights of the positive loadings of aldehydes and ketones, esters, and high alcohols were larger, and thus PC2 reflects the aroma of QN. As can be seen from Figure 4B3, BLU and WH-treated samples were in the positive direction of the PC2 axis, suggesting that they had a greater tendency to increase the aroma of QN than other treatments. The eigenvalue of PC3 was 1.664 with a variance contribution of 9.243%, in which the positive loadings of terpenes were weighted more, thus PC3 reflects the terpene aroma of QN. As can be seen from Figure S5B5, anthocyanin-related parameters were mainly concentrated in the negative half-axis of PC3, and aroma-related parameters were mainly concentrated in the positive half-axis of PC3. Among them, the parameters of aldehydes and ketones are far away from the rest of the parameters, indicating a low correlation between them and the rest of the parameters. R-B and WH-treated samples were in the positive direction of the PC3 axis, suggesting that they had a greater tendency to increase the content of terpene aroma than BLU, RE, and CK.

### 3.8. Comprehensive Evaluation of 3 Grape Varieties

The data for the 18 quality indicators of SB, XI, and QN were standardised separately, and the composite scores were calculated and ranked according to the variance contribution ratio of the 3 principal components and the affiliation function model. The coefficients (eigenvectors) corresponding to each indicator in the 3 principal components were obtained by dividing the data of each indicator in the principal component loading matrix (Table S2) by the square root of the corresponding eigenvalues of the principal components, and the score expressions for the 3 principal components were constructed using the eigenvectors as weights (Table S3). A comprehensive grape quality assessment model (Table S3) was constructed using the variance contribution ratio corresponding to the 3 principal components as weights.

The higher the composite score, the better the overall performance of the grape berries of the treatment, and the ranking of the composite score is shown in Table 2. The comprehensive quality assessment of SB grape berries treated with different wavelengths of light supplementation was ranked as R-B > RE > BLU > WH > CK. The comprehensive quality assessment of XI was ranked as R-B > BLU > RE > WH > CK. The comprehensive quality assessment of QN was ranked as R-B > BLU > RE > WH > CK. The results showed that the R-B treatment maximized the abundance of anthocyanin-related parameters (Dp, Cy, Pt, Pn, Mv, Pn-ac, Mv-ac, t-Pn-co, t-Mv-co, TAC) and sugar-related parameters (TSS, pH, glucose, fructose), and the BLU treatment maximized the abundance of aroma-related parameters (esters, terpenes, high alcohols, aldehydes and ketones).

### 3.9. Sensory Analysis of Grapes

**Figure 5:**
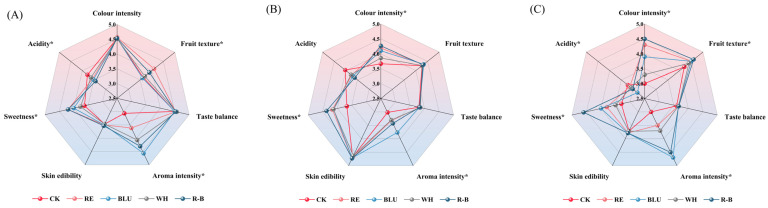
(**A**–**C**) Radar plots of sensory assessment of different treatments at harvest for SB, XI, and QN. * denotes statistical significance among treatments using one-way ANOVA (*p* < 0.05). Abbreviations: CK, no light treatment; RE, red light treatment; BLU, blue light treatment; WH, white light treatment; R-B, red and blue light treatment (1:1).

In order to understand the difference in the actual taste of berries from SB, XI, and QN treated with different wavelengths of light supplementation, this study organised a blind tasting of three table grapes treated with different wavelengths of light supplementation with the help of a trained sensory tasting panel and plotted the results of the tastings as radar charts, as shown in Figure 5.

Colour intensity, sweetness, and skin edibility scores of SB grape berries in the R-B treatment were higher than all other treatments; the aroma intensity of SB grape berries in the BLU treatment obtained significantly higher sensory scores than the other treatments; and the fruit texture scores of SB grape berries in the RE treatment were higher than the other treatments (Figure 5A).

XI under R-B treatment had the highest colour intensity, sweetness, and skin edibility compared to other treatments. Aroma intensity sensory scores of XI grape berries in BLU treatment were significantly higher than other treatments, and acidity scores were highest in CK, which was in general agreement with the results of SB (Figure 5B).

The difference in scores among the QN treatments was large, with R-B-treated berries having the highest colour intensity, sweetness, fruit texture, and skin edibility scores among the treatments, BLU-treated QN grape berries having significantly higher aroma intensity sensory scores than the other treatments, and CK having the highest acidity scores, which was in general agreement with the results of SB and XI (Figure 5C).

Overall, R-B-treated SB, XI, and QN had the highest sensory scores for colour intensity, sweetness, and skin edibility, and BLU-treated SB, XI, and QN had the highest sensory scores for aroma intensity.

## 4. Discussion

Many studies have demonstrated that the fruit quality of berry plants, such as tomatoes, blueberries, and strawberries, can be influenced by the spectral composition of the environment [37,38,39]. In a supplemental light experiment with blueberries, fruit soluble sugar content was elevated in the supplemental light treatment with a 2:1 ratio of red to blue light [38]. The soluble sugar and organic acid contents of green onion were much higher under blue-white combined light treatment [40]. Blue light treatment induced the synthesis of sucrose in mango pulp [41]. In this study, compared with other treatments, the R-B treatment had the highest berry soluble solids and hexose contents of the three grape varieties at the harvest stage, and the rest of the wavelengths of light treatments also increased the soluble sugar contents of the fruits, which is basically in agreement with the results of the previous study, suggesting that supplemental light may be favourable to the accumulation of sugars in grapes, and that the combination of red and blue light may be the most favourable light quality for the accumulation of sugars in table grape fruits. In a supplemental light test on Cabernet Sauvignon (*Vitis vinifera* L.), blue light treatments reduced the content of organic acids and increased the content of soluble sugars in the grape berries compared with white light [42]. However, blue light treatment increased the total sugar and titratable acid content of strawberries during storage [43]. In this study, all supplemental light treatments increased the pH and decreased the acid content of the three table grape berries, suggesting that there may be interspecific differences in the effects of different wavelengths of light on organic acids in grape berries.

Red light favoured the synthesis of proanthocyanidins in strawberries, and blue light accelerated the accumulation of anthocyanins [44]. The anthocyanin content of ‘Red Zaosu’ pear was elevated by 72 h of blue light treatment; however, red light treatment had no effect on it [45]. Increasing the proportion of blue light can increase the anthocyanin content of purple pepper fruits by enhancing anthocyanin biosynthesis [46]. In this study, the R-B treatment was most favourable for the accumulation of the nine major individual anthocyanins and total anthocyanin in the three table grape varieties, followed by the RE treatment, which was in general agreement with the results of previous studies, suggesting that both supplemental red and blue light are favourable for the accumulation of anthocyanins in the fruits and that R-B may be the most favourable light quality for anthocyanin accumulation.

The content of 39 volatile compounds in red pitaya changed after blue light treatment [47]. The blue light treatment significantly increased the content of volatile compounds in Cabernet Sauvignon (*Vitis vinifera* L.) compared to the white light treatment [42]. High daily light integral green light and low daily light integral red light reduced the accumulation of major aroma substances in tomato [48]. In this study, blue light was most favourable for the accumulation of volatile compounds in the berries of the three table grape varieties, which is in general agreement with previous studies, suggesting that blue light may be most favourable for the accumulation of volatile compounds in the berries of grapes compared with other light qualities.

After storing melons under different types of visible light conditions for 5 d, it was found that the highest sensory scores were obtained from melons stored under white light storage conditions [49]. Postharvest light treatments can be used to regulate the sensory quality of strawberries; the blue light-treated fruit is liked significantly more than the control [50]. Previous studies have irradiated SB grape leaves with red and blue light, as well as combined light, and found that blue light and combined red and blue light improved fruit composition [51]. In this study, the three table grape berries in the R-B treatment obtained the highest composite score on principal component analysis and also the highest sensory scores for colour intensity, sweetness, and skin edibility, and the three table grape berries in the BLU treatment obtained the highest sensory score for aroma intensity. This is basically consistent with the results of previous studies on the effect of light on grape fruit quality, but not entirely consistent with the results of studies on other crops, which may be due to interspecific differences, or because of differences in the preferences of different consumers. This suggests that when supplemental light treatments are applied to table grapes, R-B supplemental light treatments may be most favourable for the improvement of overall fruit quality, whereas BLU may be most favourable for the accumulation of volatile compounds in table grape fruit as far as fruit aroma is concerned.

## 5. Conclusions

The light environment in vineyards under protected cultivation is adversely affected by the long-term use of plastic film, and the use of LEDs of different wavelengths to supplement light on vines can improve the light environment in vineyards and enhance the quality of grapes. All four supplemental light treatments increased the content of anthocyanins, sugars, and volatile compounds in three grape varieties compared to CK. Among them, R-B was the most favourable treatment for the accumulation of sugars and anthocyanins in grapes, as well as the increase in pH. The BLU treatment was most favourable for the accumulation of volatile compounds. The grapes treated with R-B light all obtained the highest composite scores via principal component analysis. The results of the sensory analysis indicated that the highest aroma intensity scores were obtained for the three grape berries under the BLU treatment, and the highest colour intensity, sweetness, and skin edibility sensory scores were obtained for the three grape berries under the R-B treatment.

## Figures and Tables

**Table 1:** Free volatile compounds in grape berries at harvest from five treatments (μg/L).

No.	Compounds			SB					XI					QN		
		CK	RE	BLU	WH	R-B	CK	RE	BLU	WH	R-B	CK	RE	BLU	WH	R-B
	esters															
1	ethyl acetate	7308.33 ± 104.00 e	10913.81 ± 990.81 c	20479.76 ± 497.76 a	8452.09 ± 151.67 d	13776.83 ± 241.71 b	1936.84 ± 83.78 d	2496.97 ± 337.75 d	7055.03 ± 297.91 a	3121.92 ± 265.32 c	5671.48 ± 394.42 b	3088.34 ± 30.67 e	5874.37 ± 19.60 c	10001.62 ± 61.10 a	3723.10 ± 20.50 d	7264.28 ± 19.81 b
2	butanoic acid, ethyl ester	nd	nd	nd	nd	nd	nd	nd	nd	nd	nd	27.96 ± 0.24 d	52.56 ± 1.84 c	54.37 ± 2.21 bc	57.60 ± 2.07 b	83.06 ± 2.39 a
3	benzoic acid, ethyl ester	17.29 ± 0.57 a	17.53 ± 0.55 a	18.17 ± 0.58 a	17.46 ± 0.58 a	18.54 ± 1.16 a	2.61 ± 0.53 d	3.68 ± 0.42 c	6.55 ± 0.39 b	5.72 ± 0.36 b	8.69 ± 0.41 a	17.63 ± 0.57 a	17.91 ± 0.30 a	17.78 ± 0.40 a	17.95 ± 82 a	18.05 ± 0.58 a
4	hexanoic acid, ethyl ester	nd	nd	nd	nd	nd	nd	nd	nd	nd	nd	19.83 ± 1.34 e	49.22 ± 1.43 b	42.16 ± 1.51 c	39.15 ± 0.81 d	61.24 ± 1.32 a
5	2-butenoic acid, 3-methyl-, ethyl ester	nd	24.00 ± 0.33 a	24.40 ± 0.62 a	22.97 ± 1.55 a	23.48 ± 0.67 a	2.68 ± 0.41 d	5.62 ± 0.53 c	7.48 ± 0.33 b	5.56 ± 0.33 c	9.85 ± 0.24 a	nd	nd	nd	nd	nd
6	heptanoic acid, ethyl ester	nd	nd	nd	nd	nd	nd	nd	nd	nd	nd	388.14 ± 13.98 a	382.60 ± 10.09 a	382.65 ± 9.97 a	382.44 ± 10.04 a	382.36 ± 10.37 a
7	propanoic acid, 2-methyl-, anhydride	nd	nd	nd	nd	2.43 ± 0.20 a	nd	nd	nd	nd	nd	nd	nd	nd	nd	nd
8	pentanoic acid, ethyl ester	nd	nd	nd	nd	nd	nd	nd	nd	nd	nd	55.15 ± 3.44 e	233.48 ± 10.03 c	273.32 ± 9.78 b	183.42 ± 9.73 d	325.41 ± 10.12 a
9	methyl benzoate	nd	nd	nd	nd	nd	nd	8.42 ± 0.33 a	nd	8.57 ± 0.25 a	8.67 ± 0.45 a	nd	nd	nd	nd	nd
10	octanoic acid, ethyl ester	nd	nd	nd	nd	nd	nd	nd	nd	nd	nd	13.99 ± 0.41 c	15.80 ± 0.75 b	14.84 ± 0.22 bc	15.96 ± 0.80 b	17.66 ± 0.83 a
11	isophthalic acid, di(2-isopropylphenyl) ester	24.83 ± 1.70 ab	24.91 ± 0.63 ab	26.29 ± 1.28 a	23.95 ± 0.55 b	25.53 ± 0.59 ab	nd	nd	nd	nd	nd	nd	nd	nd	nd	nd
12	3-hexenoic acid, ethyl ester	nd	nd	nd	nd	nd	nd	nd	nd	nd	nd	14.11 ± 0.57 a	14.00 ± 0.26 a	14.79 ± 0.60 a	13.96 ± 0.19 a	15.00 ± 0.97 a
13	isopropyl butyrate	nd	nd	nd	nd	nd	17.07 ± 0.54 a	17.19 ± 0.72 a	17.32 ± 0.42 a	17.29 ± 0.75 a	9.36 ± 0.20 b	nd	nd	nd	nd	nd
14	pentanoic acid, 5-hydroxy-, 2,4-di-t-butylphenyl esters	nd	nd	nd	nd	nd	nd	nd	nd	nd	nd	44.85 ± 0.30 ab	36.88 ± 0.97 c	43.85 ± 0.40 b	31.99 ± 0.82 d	45.70 ± 0.60 a
15	(Z)-butanoic acid, 3-hexenyl ester	nd	nd	3.66 ± 0.20 a	3.36 ± 0.17 b	3.71 ± 0.16 a	nd	nd	nd	nd	nd	nd	nd	nd	nd	nd
16	carbonic acid, heptyl vinyl ester	nd	nd	nd	nd	nd	nd	nd	nd	nd	nd	19.68 ± 0.54 a	19.91 ± 0.94 a	19.82 ± 0.51 a	19.81 ± 0.28 a	20.77 ± 0.60 a
17	hexanoic acid, ethyl ester	nd	nd	nd	nd	nd	13.38 ± 0.52 a	13.02 ± 0.72 a	12.92 ± 0.76 a	13.14 ± 0.99 a	7.21 ± 0.19 b	nd	nd	nd	nd	nd
18	(E)-2-butenoic acid, ethyl ester	nd	nd	nd	nd	nd	nd	nd	nd	nd	nd	20.87 ± 0.61 d	24.84 ± 0.43 b	23.12 ± 0.66 c	25.79 ± 0.58 b	27.98 ± 0.86 a
19	(Z)-3-hexen-1-ol, formate	3.53 ± 0.15 b	3.55 ± 0.10 b	3.81 ± 0.10 a	3.50 ± 0.16 b	3.88 ± 0.05 a	nd	nd	nd	nd	nd	nd	nd	nd	nd	nd
20	butanoic acid, 3-hydroxy-, ethyl ester	nd	nd	nd	nd	nd	nd	nd	nd	nd	nd	19.25 ± 0.21 c	20.49 ± 0.21 a	19.67 ± 0.04 b	20.28 ± 0.19 a	20.42 ± 0.26 a
21	α-methyl-benzenemethanol	nd	nd	nd	nd	nd	23.81 ± 0.70 a	23.83 ± 0.71 a	23.92 ± 0.91 a	12.10 ± 0.59 b	12.83 ± 0.60 b	nd	nd	nd	nd	nd
22	2-hexenoic acid, ethyl ester	nd	nd	nd	nd	nd	nd	nd	nd	nd	nd	13.06 ± 0.75 c	15.28 ± 0.38 ab	14.22 ± 0.60 bc	15.36 ± 0.55 ab	16.08 ± 0.77 a
23	1,3-benzenediol, monobenzoate	3.57 ± 0.15 b	4.44 ± 0.06 a	4.23 ± 0.19 a	4.20 ± 0.28 a	4.53 ± 0.43 a	nd	nd	nd	nd	nd	nd	nd	nd	nd	nd
	subtotal	7357.55 ± 102.98 e	10988.25 ± 990.16 c	20560.32 ± 495.95 a	8527.52 ± 152.56 d	13858.68 ± 241.96 b	1996.38 ± 84.30 d	2568.73 ± 337.43 d	7123.23 ± 298.70 a	3184.31 ± 265.74 c	5728.08 ± 395.00 b	3742.87 ± 41.76 e	6757.34 ± 21.89 c	10922.22 ± 68.04 a	4546.81 ± 17.21 d	8298.02 ± 21.55 b
	Proportion (%)	39.31	41.65	50.35	34.17	42.51	17.86	19.15	35.15	24.63	32.55	24.55	34.09	40.25	25.36	36.42
	terpenes															
24	linalool	9.41 ± 0.31 c	9.44 ± 0.40 c	12.48 ± 1.40 a	10.10 ± 0.51 bc	11.39 ± 0.26 ab	9.21 ± 0.29 a	9.48 ± 0.39 a	9.66 ± 0.22 a	9.22 ± 0.19 a	9.59 ± 0.44 a	1.57 ± 0.23 d	3.15 ± 0.18 c	9.20 ± 0.26 a	3.38 ± 0.31 c	7.65 ± 0.14 b
25	γ-terpinene	1.65 ± 0.21 e	225.53 ± 20.98 c	309.38 ± 2.85 a	250.39 ± 2.36 b	168.89 ± 2.03 d	3.52 ± 0.43 c	3.54 ± 0.18 c	265.44 ± 0.44 b	0.54 ± 0.41 c	487.09 ± 53.81 a	nd	nd	nd	nd	nd
26	α-terpinene	45.82 ± 3.08 e	77.47 ± 2.82 d	143.54 ± 2.14 a	113.68 ± 2.54 b	106.17 ± 3.97 c	nd	nd	nd	nd	nd	nd	nd	nd	nd	nd
27	d-limonene	21.93 ± 0.83 c	34.20 ± 0.49 b	47.94 ± 1.27 a	44.42 ± 3.84 a	32.27 ± 1.78 b	nd	nd	nd	nd	nd	2.59 ± 0.41 e	7.33 ± 0.29 d	14.36 ± 0.44 a	9.59 ± 0.41 c	12.03 ± 0.77 b
28	α-terpineol	nd	2.52 ± 0.22 c	4.35 ± 0.33 a	3.56 ± 0.46 b	4.56 ± 0.40 a	nd	nd	5.30 ± 0.31 a	0.82 ± 0.14 b	nd	nd	nd	nd	nd	nd
29	(R)-4-methyl-1-(1-methylethyl)-3-cyclohexen-1-ol	nd	6.61 ± 0.39 c	9.44 ± 0.42 a	9.52 ± 0.32 a	8.50 ± 0.15 b	nd	nd	nd	nd	nd	nd	nd	nd	nd	nd
30	3-methyl-6-(1-methylethylidene)-cyclohexene	nd	nd	nd	nd	nd	7.47 ± 0.42 b	3.80 ± 0.04 c	61.49 ± 0.68 a	3.58 ± 0.07 c	2.76 ± 0.17 d	nd	nd	nd	nd	nd
31	(S)-3,7-dimethyl-7-octen-1-ol	0.88 ± 0.09 e	9.34 ± 0.37 c	10.66 ± 0.21 b	8.47 ± 0.44 d	12.55 ± 0.47 a	nd	nd	nd	nd	nd	nd	nd	nd	nd	nd
32	geraniol	266.64 ± 3.57 e	333.38 ± 18.30 d	487.81 ± 11.91 b	388.79 ± 10.06 c	679.00 ± 7.19 a	6.50 ± 0.44 d	17.12 ± 0.89 c	26.54 ± 1.31 b	31.40 ± 0.63 a	17.53 ± 1.33 c	4.73 ± 0.27 c	4.35 ± 0.17 d	5.28 ± 0.13 b	4.10 ± 0.08 d	5.62 ± 0.14 a
33	(Z)-3,7-dimethyl-2,6-octadien-1-ol	38.56 ± 0.62 d	183.78 ± 7.2 c	271.82 ± 8.19 b	189.66 ± 3.25 c	354.66 ± 10.10 a	nd	nd	nd	nd	nd	nd	nd	nd	nd	nd
34	1,3,8-p-menthatriene	69.65 ± 1.77 e	144.36 ± 3.39 d	195.76 ± 0.14 b	204.82 ± 2.97 a	164.08 ± 3.42 c	nd	nd	nd	nd	nd	nd	nd	nd	nd	nd
35	trifluoroacetyl-lavandulol	nd	nd	nd	nd	nd	4.49 ± 0.38 c	4.54 ± 0.43 c	8.59 ± 0.28 b	9.32 ± 0.07 a	4.21 ± 0.17 c	nd	nd	nd	nd	nd
36	styrene	nd	nd	nd	nd	nd	nd	nd	nd	nd	nd	239.19 ± 5.11 b	170.78 ± 2.77 c	178.07 ± 5.11 c	168.46 ± 2.46 c	357.41 ± 13.44 a
37	3,4-dimethyl-1,5-heptadiene	nd	nd	nd	nd	nd	nd	nd	nd	nd	nd	3628.71 ± 15.30 b	3462.72 ± 9.94 d	3580.78 ± 10.18 c	3787.61 ± 10.12 a	3568.43 ± 9.87 c
	subtotal	454.54 ± 6.16 e	1026.63 ± 23.53 d	1493.18 ± 18.83 b	1223.42 ± 16.27 c	1542.08 ± 13.98 a	31.18 ± 1.31 c	38.48 ± 1.36 c	377.02 ± 1.38 b	54.88 ± 0.62 c	521.20 ± 53.61 a	3876.79 ± 20.79 b	3648.33 ± 7.85 d	3787.68 ± 13.96 c	3973.14 ± 12.53 a	3951.15 ± 9.83 a
	Proportion (%)	2.43	3.89	3.66	4.90	4.73	0.28	0.29	1.86	0.42	2.96	25.43	18.40	13.96	22.16	17.34
	high alcohols															
38	2-ethyl-1-hexanol	37.63 ± 0.97 bc	40.15 ± 2.09 b	54.27 ± 1.20 a	22.11 ± 0.85 d	35.54 ± 0.78 c	22.67 ± 0.77 c	27.63 ± 1.50 b	33.68 ± 1.19 a	29.60 ± 1.06 b	33.57 ± 0.75 a	20.77 ± 0.38 b	18.36 ± 0.54 c	26.95 ± 0.95 a	21.07 ± 0.84 b	27.30 ± 2.16 a
39	cyclohexanol	15.66 ± 0.96 e	19.38 ± 0.44 d	42.84 ± 0.26 a	28.73 ± 1.70 b	25.28 ± 0.69 c	nd	nd	nd	nd	nd	nd	nd	nd	nd	nd
40	1-hexanol	131.59 ± 1.93 d	157.76 ± 1.41 c	232.31 ± 2.21 a	103.83 ± 3.52 e	162.65 ± 1.35 b	19.79 ± 1.01 d	28.80 ± 1.03 c	37.75 ± 1.15 a	32.47 ± 1.06 b	30.64 ± 0.97 bc	33.31 ± 1.61 c	37.23 ± 1.80 b	48.53 ± 0.59 a	47.60 ± 1.03 a	38.19 ± 0.47 b
41	2-buten-1-ol	3.52 ± 0.37 b	2.62 ± 0.36 c	3.45 ± 0.20 b	5.52 ± 0.44 a	5.74 ± 0.35 a	0.34 ± 0.11 c	2.44 ± 0.02 b	2.40 ± 0.18 b	5.22 ± 0.08 a	2.27 ± 0.23 b	0.52 ± 0.40 b	0.77 ± 0.18 b	1.60 ± 0.33 a	0.66 ± 0.32 b	0.67 ± 0.31 b
42	2-(4-methylphenyl)propan-2-ol	3.48 ± 0.32 d	12.33 ± 1.10 c	25.27 ± 1.17 a	18.58 ± 1.17 b	17.85 ± 0.42 b	nd	nd	nd	nd	nd	13.61 ± 0.61 d	14.05 ± 0.27 d	54.90 ± 1.61 a	34.27 ± 2.37 c	46.06 ± 1.51 b
43	phenylethyl alcohol	32.84 ± 1.50 c	66.69 ± 1.26 b	63.60 ± 0.81 b	65.33 ± 1.98 b	105.53 ± 3.22 a	nd	nd	nd	nd	nd	nd	nd	nd	nd	nd
44	1-octanol	64.22 ± 2.32 c	69.34 ± 0.37 b	75.99 ± 1.67 a	65.14 ± 2.51 c	72.55 ± 0.47 b	32.50 ± 1.99 b	31.72 ± 0.24 b	42.88 ± 1.88 a	41.53 ± 0.41 a	40.81 ± 1.85 a	93.78 ± 1.61 b	94.91 ± 0.66 b	113.83 ± 1.11 a	93.94 ± 0.27 b	94.51 ± 0.48 b
45	isohexanol	6.66 ± 0.36 c	7.67 ± 0.34 b	9.45 ± 0.52 a	7.55 ± 0.47 b	7.57 ± 0.33 b	6.33 ± 0.30 c	7.72 ± 0.24 b	8.62 ± 0.31 a	7.61 ± 0.52 b	7.58 ± 0.38 b	6.38 ± 0.12 c	7.46 ± 0.30 b	8.64 ± 0.24 a	8.39 ± 0.38 a	7.41 ± 0.26 b
46	(E)-2-hexen-1-ol	18.52 ± 1.10 b	21.38 ± 0.72 b	28.85 ± 2.14 a	18.97 ± 0.33 b	28.13 ± 2.77 a	73.80 ± 3.57 a	72.51 ± 2.28 a	66.59 ± 0.71 b	61.75 ± 0.32 c	72.71 ± 0.59 a	63.84 ± 3.49 cd	67.85 ± 0.52 bc	72.68 ± 1.69 a	61.52 ± 2.00 d	70.85 ± 1.13 ab
47	1-decanol	18.65 ± 1.11 ab	16.69 ± 2.04 b	20.10 ± 0.70 a	20.49 ± 0.19 a	16.75 ± 0.52 b	18.50 ± 0.52 c	18.20 ± 0.38 c	20.92 ± 0.23 b	19.73 ± 1.15 bc	22.56 ± 1.14 a	18.90 ± 0.19 bc	19.32 ± 0.22 ab	20.59 ± 0.36 ab	16.84 ± 2.33 c	21.07 ± 0.32 a
48	2-heptanol	93.74 ± 1.77 c	97.78 ± 1.21 bc	117.48 ± 5.76 a	101.10 ± 9.67 bc	105.06 ± 4.60 b	91.89 ± 0.15 b	92.24 ± 0.78 b	113.00 ± 7.48 a	104.72 ± 5.41 a	107.41 ± 1.55 a	34.49 ± 1.25 c	35.00 ± 0.37 bc	46.42 ± 1.44 a	38.33 ± 0.90 b	43.12 ± 3.45 a
49	isoamylol	2145.66 ± 30.94 e	3072.71 ± 50.00 c	4789.51 ± 47.12 a	2751.06 ± 44.63 d	4091.95 ± 57.76 b	2132.53 ± 18.45 e	3183.00 ± 10.68 c	4853.65 ± 9.66 a	2248.18 ± 13.11 d	3660.53 ± 27.17 b	2137.31 ± 33.02 e	3184.89 ± 27.27 c	4850.34 ± 16.38 a	2879.77 ± 9.92 d	4322.11 ± 58.41 b
50	(E)-3-hexen-1-ol	139.16 ± 11.98 ab	130.80 ± 3.18 b	143.87 ± 3.42 a	113.68 ± 2.54 c	106.17 ± 3.97 c	122.34 ± 2.98 d	130.34 ± 0.30 c	146.42 ± 1.00 a	125.39 ± 1.73 d	136.36 ± 2.91 b	125.12 ± 4.54 c	124.28 ± 4.75 c	146.93 ± 1.32 a	125.73 ± 4.32 c	134.52 ± 3.86 b
51	(Z)-3-hexen-1-ol	71.93 ± 1.17 a	62.87 ± 1.88 c	67.94 ± 1.27 ab	64.42 ± 3.84 bc	64.27 ± 2.92 bc	60.37 ± 2.57 b	60.84 ± 2.15 b	73.05 ± 1.42 a	52.91 ± 0.98 c	70.01 ± 1.84 a	75.14 ± 3.87 a	62.52 ± 0.03 c	68.49 ± 1.66 b	78.79 ± 1.15 a	70.10 ± 3.24 b
52	(E)-2-hexen-1-ol	34.15 ± 3.34 c	42.33 ± 1.10 b	45.27 ± 1.17 ab	48.58 ± 1.17 a	47.18 ± 1.18 a	16.99 ± 0.80 e	22.75 ± 1.93 c	31.84 ± 0.47 a	20.33 ± 1.36 d	27.07 ± 0.44 b	16.26 ± 1.55 c	19.35 ± 0.81 c	32.88 ± 2.53 b	31.49 ± 1.56 b	37.43 ± 2.23 a
53	(Z)-2-hexen-1-ol	nd	56.69 ± 1.26 b	55.60 ± 2.24 b	65.33 ± 1.98 b	55.53 ± 3.22 a	7.44 ± 0.36 b	6.75 ± 0.32 b	52.22 ± 0.71 a	52.24 ± 0.78 a	47.23 ± 5.95 a	6.83 ± 0.13 c	7.66 ± 0.15 c	13.62 ± 2.26 b	34.42 ± 2.33 a	11.63 ± 1.34 b
54	1-pentanol	1289.84 ± 3.34 b	1196.88 ± 63.48 c	1369.98 ± 5.76 a	1253.47 ± 10.31 bc	1276.23 ± 16.32 b	1278.09 ± 13.60 c	1327.20 ± 14.81 b	1375.67 ± 2.74 a	1340.01 ± 6.31 b	1332.63 ± 14.15 b	1292.82 ± 5.36 bc	1321.24 ± 5.69 b	1373.09 ± 6.61 a	1271.72 ± 35.00 c	1261.41 ± 11.60 c
55	2-pentanol	1247.58 ± 20.07 d	1382.34 ± 14.88 b	1510.51 ± 8.78 a	1130.70 ± 15.37 e	1321.44 ± 9.99 c	1266.38 ± 34.18 e	1375.91 ± 15.48 c	1537.04 ± 25.54 a	1333.67 ± 6.01 d	1447.19 ± 12.14 b	1236.12 ± 8.53 b	1239.27 ± 3.71 b	1452.41 ± 49.82 a	1422.83 ± 17.78 a	1410.76 ± 4.73 a
56	3-pentanol	471.65 ± 0.21 ab	425.53 ± 20.98 d	489.38 ± 7.16 a	450.39 ± 2.36 c	468.89 ± 2.03 bc	474.82 ± 5.91 c	489.01 ± 5.90 ab	495.83 ± 2.26 a	479.39 ± 7.86 bc	482.41 ± 7.05 bc	412.67 ± 1.00 d	486.34 ± 4.66 b	515.96 ± 4.65 a	315.00 ± 8.14 e	468.96 ± 1.61 c
57	benzalcohol	371.62 ± 8.00 d	508.42 ± 4.90 b	649.45 ± 3.24 a	474.39 ± 7.65 c	482.46 ± 9.91 c	361.21 ± 1.03 e	521.67 ± 2.02 c	652.08 ± 0.84 a	458.10 ± 15.09 d	607.94 ± 6.71 b	469.24 ± 2.55 c	469.63 ± 0.84 c	497.43 ± 1.54 a	476.84 ± 2.33 b	477.73 ± 6.08 b
58	1-octen-3-ol	441.24 ± 26.20 c	483.11 ± 7.28 b	533.50 ± 15.36 a	442.89 ± 15.08 c	417.44 ± 10.01 c	412.89 ± 3.08 e	465.03 ± 5.66 c	513.85 ± 2.69 a	432.91 ± 9.34 d	481.34 ± 9.62 b	364.20 ± 1.62 d	539.79 ± 14.43 c	650.65 ± 15.41 a	516.94 ± 15.79 c	614.51 ± 9.90 b
	subtotal	6639.35 ± 91.17 e	7873.47 ± 77.50 c	10,328.63 ± 40.14 a	7252.27 ± 40.48 d	8914.21 ± 84.61 b	6398.87 ± 34.42 e	7863.77 ± 39.19 c	10,057.49 ± 27.74 a	6845.76 ± 21.41 d	8610.27 ± 44.59 b	6421.31 ± 40.62 e	7749.92 ± 34.34 c	9995.94 ± 43.37 a	7476.17 ± 64.95 d	9158.35 ± 60.32 b
	Proportion (%)	35.47	29.84	25.29	29.05	27.34	57.25	58.62	49.63	52.94	48.92	42.12	39.09	36.84	41.70	40.20
	aldehydes and ketones															
59	hexanal	371.20 ± 8.07 e	809.12 ± 5.04 a	635.66 ± 9.95 c	473.42 ± 5.78 d	677.01 ± 10.09 b	260.54 ± 2.81 a	241.52 ± 1.71 b	183.55 ± 2.69 d	231.77 ± 3.02 c	143.30 ± 2.98 e	174.28 ± 10.29 d	202.23 ± 2.01 c	376.19 ± 3.17 a	241.68 ± 6.25 b	205.46 ± 7.66 c
60	(E,E)-2,4-hexadienal	1.51 ± 0.42 c	3.55 ± 0.44 a	3.32 ± 0.08 a	3.47 ± 0.31 a	2.42 ± 0.19 b	1.42 ± 0.31 a	1.71 ± 0.25 a	1.46 ± 0.22 a	1.49 ± 0.21 a	0.70 ± 0.30 b	nd	nd	nd	nd	nd
61	2-hexenal	3864.78 ± 20.79 e	5546.80 ± 32.21 d	7648.19 ± 25.26 a	7320.50 ± 15.93 c	7418.99 ± 8.98 b	2397.41 ± 20.00 c	2604.08 ± 27.00 a	2414.04 ± 15.11 c	2518.62 ± 31.98 b	2493.10 ± 15.18 b	917.50 ± 1.58 e	1318.75 ± 15.33 c	1928.01 ± 11.29 a	1554.82 ± 20.76 b	1014.66 ± 10.21 d
62	2,4-dihydroxybenzaldehyde	3.62 ± 0.23 b	3.42 ± 0.51 b	6.45 ± 0.36 a	2.72 ± 0.19 c	2.46 ± 0.17 c	1.68 ± 0.36 b	1.64 ± 0.29 b	3.35 ± 0.32 a	1.61 ± 0.42 b	1.39 ± 0.53 b	3.43 ± 0.32 d	4.11 ± 0.16 bc	5.26 ± 0.22 a	4.47 ± 0.36 b	3.63 ± 0.21 cd
63	benzeneacetaldehyde	1.24 ± 0.27 e	4.44 ± 0.29 d	5.50 ± 0.39 c	6.23 ± 0.22 b	7.44 ± 0.26 a	1.56 ± 0.43 c	1.60 ± 0.31 bc	1.83 ± 0.15 bc	2.27 ± 0.29 b	3.52 ± 0.47 a	nd	nd	nd	nd	nd
64	isophthalaldehyde	nd	nd	nd	nd	nd	1.66 ± 0.26 b	2.47 ± 0.39 a	2.45 ± 0.40 a	2.63 ± 0.14 a	2.25 ± 0.17 ab	nd	nd	nd	nd	nd
65	furfural	0.20 ± 0.01 e	0.34 ± 0.06 d	0.81 ± 0.02 a	0.61 ± 0.02 c	0.71 ± 0.09 b	nd	nd	nd	nd	nd	0.10 ± 0.03 d	0.33 ± 0.02 a	0.13 ± 0.02 c	0.15 ± 0.01 c	0.27 ± 0.02 b
66	1-(2,4-dimethylphenyl)-ethanone	nd	nd	nd	nd	nd	1.57 ± 0.40 b	2.42 ± 0.40 a	2.50 ± 0.20 a	2.48 ± 0.17 a	2.53 ± 0.42 a	nd	nd	nd	nd	nd
67	3,3-dimethyl-2,4-pentane dione	0.72 ± 0.03 c	1.35 ± 0.20 b	1.26 ± 0.26 b	1.58 ± 0.43 ab	1.88 ± 0.05 a	nd	nd	nd	nd	nd	nd	nd	nd	nd	nd
68	2-methyl-cyclobutanone	nd	nd	nd	nd	nd	0.55 ± 0.12 a	0.43 ± 0.37 a	0.39 ± 0.29 a	0.44 ± 0.26 a	0.58 ± 0.31 a	nd	nd	nd	nd	nd
69	2-acetyl-resorcinol	0	0.42 ± 0.35 cd	0.93 ± 0.05 ab	1.41 ± 0.43 a	0.76 ± 0.12 bc	nd	nd	nd	nd	nd	nd	nd	nd	nd	nd
70	nonanal	nd	nd	nd	nd	nd	nd	nd	nd	nd	2.52 ± 0.28 a	nd	nd	nd	nd	nd
71	9,10-anthracenedione	6.68 ± 0.33 c	8.30 ± 0.33 b	27.96 ± 1.51 a	7.50 ± 0.37 bc	7.31 ± 0.19 bc	nd	nd	nd	nd	nd	nd	nd	nd	nd	nd
72	1-octen-3-one	0.43 ± 0.30 b	1.61 ± 0.29 a	1.75 ± 0.18 a	0.62 ± 0.25 b	1.61 ± 0.32 a	nd	nd	nd	nd	nd	nd	nd	nd	nd	nd
73	4-methyl benzene pentanone	0.74 ± 0.05 e	8.52 ± 0.34 d	12.75 ± 0.45 a	11.35 ± 0.21 b	9.66 ± 0.19 c	nd	nd	nd	nd	nd	0.51 ± 0.02 c	1.23 ± 0.03 a	1.24 ± 0.05 a	0.27 ± 0.03 d	0.80 ± 0.02 b
	subtotal	4251.13 ± 22.46 e	6387.85 ± 33.10 d	8344.58 ± 15.46 a	7829.42 ± 20.88 c	8130.24 ± 11.25 b	2666.38 ± 23.02 c	2855.87 ± 27.94 a	2609.58 ± 16.26 d	2761.31 ± 31.52 b	2649.90 ± 17.73 cd	1095.82 ± 11.73 e	1526.66 ± 17.32 c	2310.83 ± 8.56 a	1801.38 ± 25.15 b	1224.81 ± 16.04 d
	Proportion (%)	22.71	24.21	20.43	31.37	24.93	23.86	21.29	12.88	21.35	15.06	7.19	7.70	8.52	10.05	5.38
	others															
74	2-oxopentanedioic acid	13.84 ± 0.95 d	17.75 ± 1.14 c	25.39 ± 1.35 b	34.05 ± 1.75 a	34.52 ± 3.11 a	18.98 ± 1.35 ab	17.69 ± 0.82 b	20.43 ± 0.59 a	19.15 ± 1.57 ab	17.08 ± 0.48 b	nd	nd	nd	nd	nd
75	(2S,4R)-4-methyl-2-tetrahydro-2H-pyran	0	87.32 ± 3.24 b	72.65 ± 2.29 c	85.92 ± 1.21 b	108.71 ± 5.27 a	nd	nd	nd	nd	nd	nd	nd	nd	nd	nd
76	3,6-dihydro-4-methyl-2-(2-methyl-1-propenyl)-2H-pyran	1.45 ± 0.31 d	2.36 ± 0.33 d	7.50 ± 0.20 b	6.47 ± 0.39 c	15.27 ± 0.91 a	nd	nd	nd	nd	nd	nd	nd	nd	nd	nd
77	2-methyl-hexanoic acid	nd	nd	nd	nd	nd	65.22 ± 2.15 d	69.68 ± 0.47 c	76.66 ± 0.98 a	65.14 ± 2.51 d	73.21 ± 1.18 b	50.20 ± 1.42 d	87.25 ± 2.13 a	53.63 ± 2.34 cd	57.14 ± 2.40 c	77.08 ± 1.91 b
78	pentanoic acid	nd	nd	nd	nd	nd	nd	nd	nd	nd	nd	53.00 ± 1.31 b	44.88 ± 0.99 c	52.74 ± 1.12 b	62.28 ± 2.12 a	64.97 ± 2.45 a
79	2-ethyl-furan	nd	nd	nd	nd	nd	nd	nd	nd	nd	nd	6.47 ± 0.10 c	9.30 ± 0.19 b	12.63 ± 1.42 a	9.66 ± 0.33 b	7.65 ± 0.24 c
	subtotal	15.29 ± 1.24 d	107.43 ± 3.35 c	105.54 ± 3.70 c	126.44 ± 3.08 b	158.50 ± 5.41 a	84.20 ± 1.45 c	87.37 ± 1.13 bc	97.09 ± 1.40 a	84.29 ± 3.31 c	90.30 ± 1.60 b	109.67 ± 2.83 e	141.43 ± 2.29 b	119.00 ± 0.60 d	129.09 ± 0.50 c	149.70 ± 1.78 a
	Proportion (%)	0.08	0.41	0.26	0.51	0.49	0.75	0.65	0.48	0.65	0.51	0.72	0.71	0.44	0.72	0.66
	total	18,717.86 ± 212.98 e	26,383.62 ± 903.44 c	40,832.25 ± 465.63 a	24,959.07 ± 207.44 d	32,603.71 ± 239.69 b	11,177.01 ± 115.53 d	13,414.23 ± 317.94 c	20,264.41 ± 313.26 a	12,930.56 ± 232.81 c	17,599.75 ± 434.81 b	15,246.45 ± 110.83 e	19,823.67 ± 46.68 c	27,135.67 ± 119.47 a	17,926.58 ± 92.54 d	22,782.03 ± 51.16 b

Note: CK: no light treatment; RE: red light treatment; BLU: blue light treatment; WH: white light treatment; R-B: red and blue light treatment (1:1). Concentrations of volatile compounds were quantified by curves of chromatographically pure standards and expressed as the means ± SD (*n* = 3); nd, not detected; different lowercase letters (a–e) in the same row indicate significant differences among five treatments through one-way ANOVA (*p* < 0.05).

**Table 2:** Principal component scores, comprehensive scores and ranking of comprehensive quality assessment.

Variety	Treatments	Factor Score			Comprehensive Score	Rank
		f1	f2	f3		
SB	CK	−4.973	−1.587	—	−3.848	5
	RE	1.469	−1.104	—	0.800	2
	BLU	−0.767	3.326	—	0.171	3
	WH	−0.528	0.126	—	−0.346	4
	R-B	4.800	−0.761	—	3.223	1
XI	CK	−3.853	0.292	−2.192	−2.772	5
	RE	−0.860	−1.241	1.316	−0.598	3
	BLU	1.655	3.003	0.628	1.707	2
	WH	−1.937	−0.772	1.384	−1.208	4
	R-B	4.994	−1.282	−1.136	2.872	1
QN	CK	−4.574	−1.160	−0.166	−3.571	5
	RE	0.876	−0.209	−2.061	0.427	3
	BLU	1.266	2.592	0.097	1.328	2
	WH	−2.459	0.330	1.292	−1.651	4
	R-B	4.891	−1.552	0.838	3.467	1

Note: f1, f2, and f3 represent the factor scores of the three principal components (PC1, PC2, and PC3). — denotes that the factor score is not taken into account. Abbreviations: CK, no light treatment; RE, red light treatment; BLU, blue light treatment; WH, white light treatment; R-B, red and blue light treatment (1:1).

## Data Availability

Data are contained within the article or Supplementary Material.

## Supplementary Materials

The following supporting information can be downloaded at: https://www.mdpi.com/article/10.3390/foods12224165/s1, Figure S1: Light intensity inside and outside the greenhouse and inside the canopy before and after supplemental light over a 20 h period; Figure S2: Content of nine individual anthocyanins in SB berries treated with different light quality supplementation at five phenological stages; Figure S3: Content of nine individual anthocyanins in XI berries treated with different light quality supplementation at five phenological stages; Figure S4: Content of nine individual anthocyanins in QN berries treated with different light quality supplementation at five phenological stages; Figure S5: Loading values of the grape quality indicators in the two PCs and the distribution of different treatments in XI (B4) and QN (B5); Table S1: Eigenvalues, variance contribution rates and cumulative contribution rates of principal components; Table S2: Loading matrix of principal components; Table S3: Score expressions of the 2 principal components and a model for comprehensive evaluation of grape quality.
