# Supplementary material for: Effect of Supplementary Light with Different Wavelengths on Anthocyanin Composition, Sugar Accumulation and Volatile Compound Profiles of Grapes
Source: Foods. 2023 Nov 17;12(22):4165. doi: 10.3390/foods12224165 (PMC10670164; doi:10.3390/foods12224165)
Supplement: Supplementary file 1 [file foods-12-04165-s001.zip › foods-2691805-supplementary.pdf]

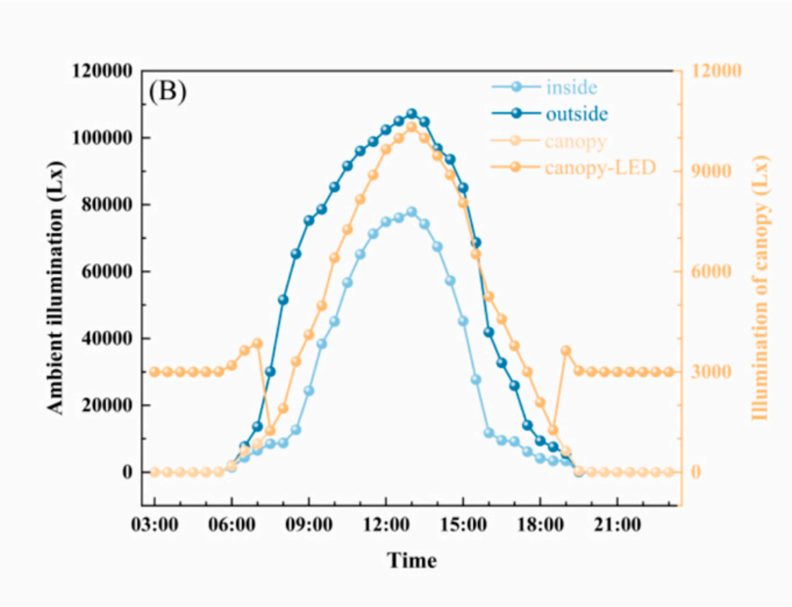

Figure S1 Light intensity inside and outside the greenhouse and inside the canopy before and after supplemental light over a 20 hour period. Inside denotes light intensity inside the greenhouse; Outside denotes light intensity outside the greenhouse; Canopy denotes light intensity inside the canopy when no LED supplemental light was used; Canopy-LED denotes light intensity inside the canopy after using LED supplemental light.

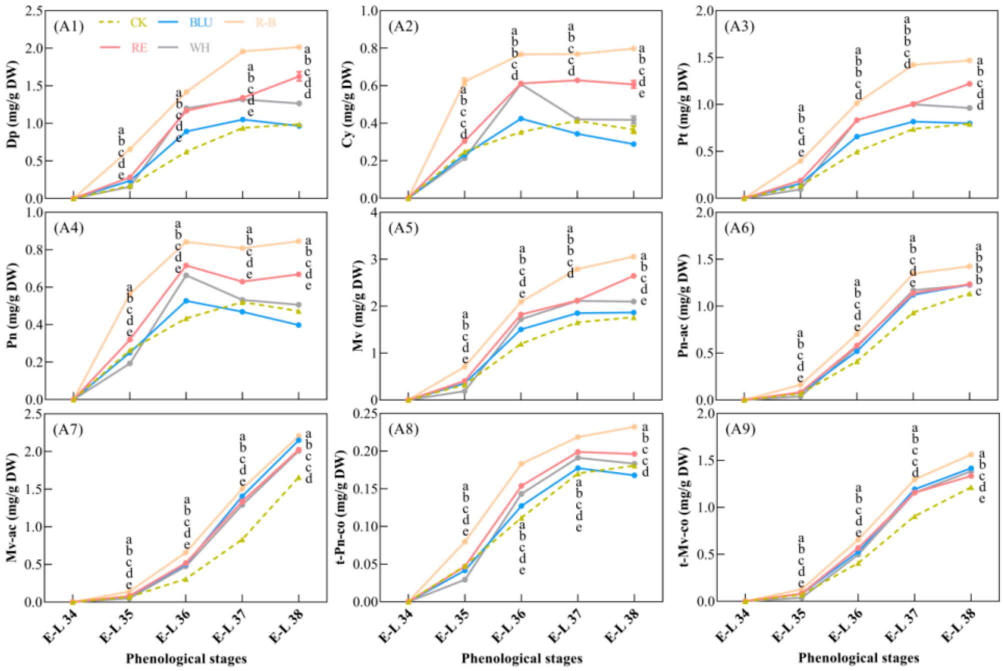

Figure S2 Content of nine individual anthocyanins in SB berries treated with different light quality supplementation at five phenological stages. Error bars are the standard deviation of the mean (n = 3). Different letters indicate significant differences among treatments using Duncan's test ($P < 0.05$).

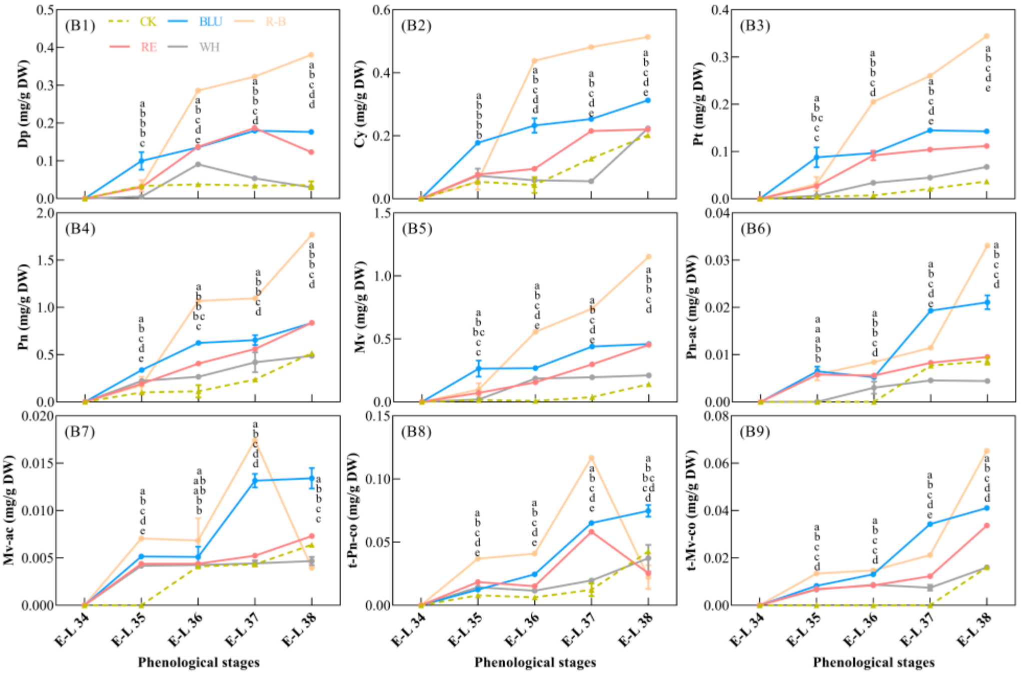

Figure S3 Content of nine individual anthocyanins in XI berries treated with different light quality supplementation at five phenological stages. Error bars are the standard deviation of the mean (n = 3). Different letters indicate significant differences among treatments using Duncan's test ($P < 0.05$).

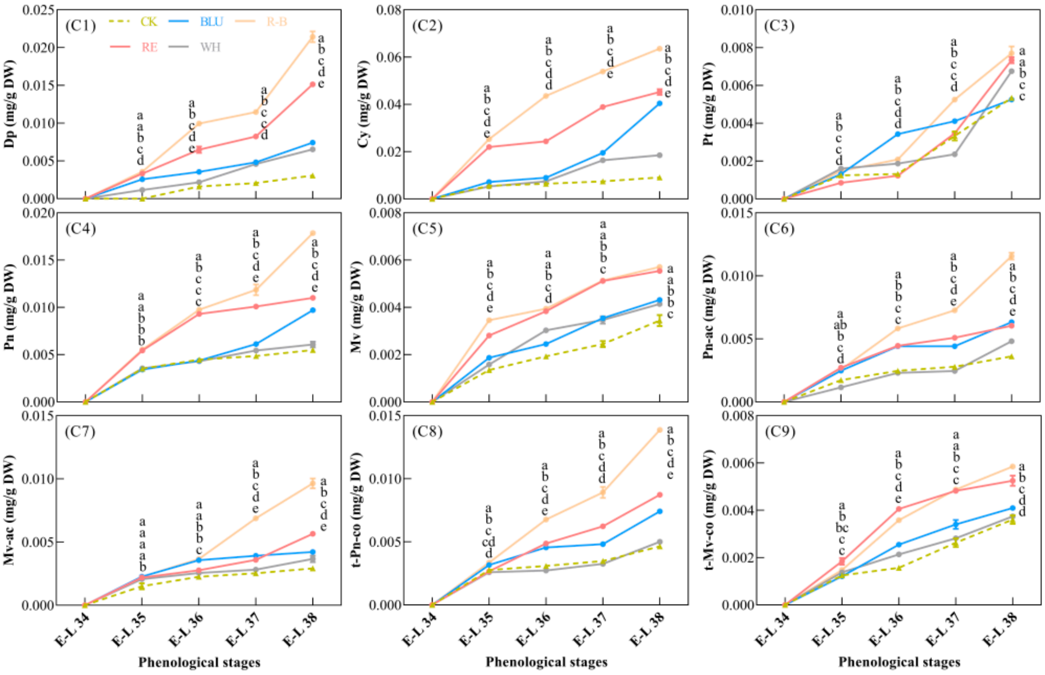

Figure S4 Content of nine individual anthocyanins in QN berries treated with different light quality supplementation at five phenological stages. Error bars are the standard deviation of the mean (n = 3). Different letters indicate significant differences among treatments using Duncan's test ($P < 0.05$).

(B4) (B5)

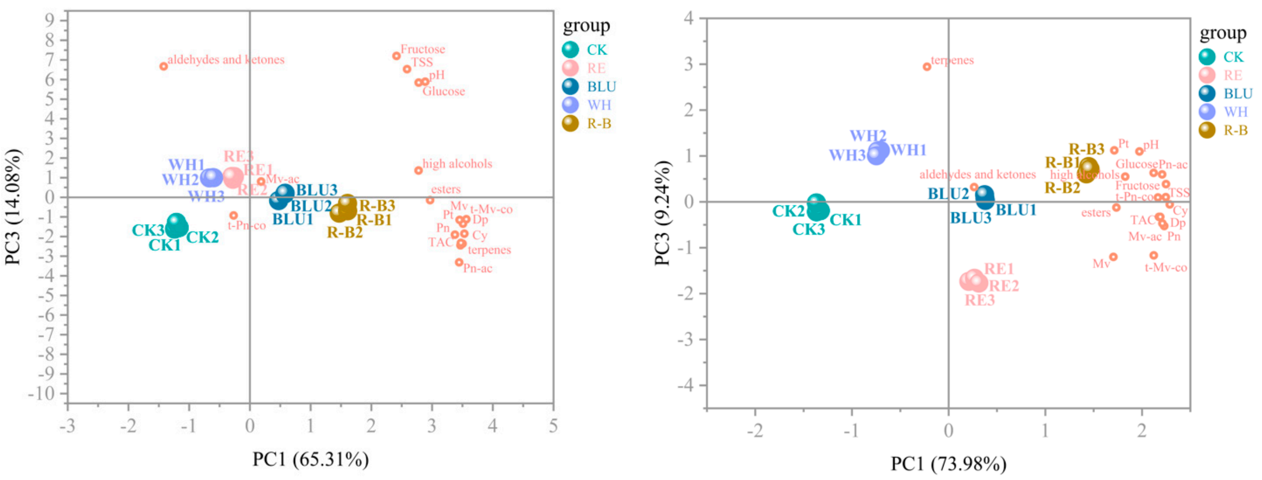

Figure S5 Loading values of the grape quality indicators in the two PCs and the distribution of different treatments in XI (B4) and QN (B5).

Table S1. Eigenvalues, variance contribution rates and cumulative contribution rates of principal components.

Variety	Principal component	Eigenvalues	Eigenvalues	Cumulative contribution rates/%
SB	PC1	12.699	70.551	70.551
	PC2	3.849	21.385	91.936
	PC3	0.967	5.371	97.307
	PC4	0.485	2.693	100.000
	PC5	6.966E-16	3.870E-15	100.000
	PC6	3.259E-16	1.810E-15	100.000
	PC7	2.629E-16	1.461E-15	100.000
	PC8	2.144E-16	1.191E-15	100.000
	PC9	1.236E-16	6.864E-16	100.000
	PC10	5.403E-17	3.002E-16	100.000
	PC11	7.761E-18	4.312E-17	100.000
	PC12	-6.704E-17	-3.724E-16	100.000
	PC13	-9.239E-17	-5.133E-16	100.000
	PC14	-1.732E-16	-9.624E-16	100.000
	PC15	-2.418E-16	-1.343E-15	100.000
	PC16	-3.106E-16	-1.726E-15	100.000
	PC17	-4.992E-16	-2.773E-15	100.000
	PC18	-5.696E-16	-3.164E-15	100.000
XI	PC1	11.755	65.305	65.305
	PC2	3.221	17.892	83.196
	PC3	2.534	14.079	97.276
	PC4	0.490	2.724	100.000

	PC5	5.955E-16	3.308E-15	100.000
	PC6	4.837E-16	2.687E-15	100.000
	PC7	3.309E-16	1.838E-15	100.000
	PC8	2.194E-16	1.219E-15	100.000
	PC9	1.172E-16	6.512E-16	100.000
	PC10	1.901E-17	1.056E-16	100.000
	PC11	2.132E-18	1.185E-17	100.000
	PC12	-5.219E-17	-2.900E-16	100.000
	PC13	-1.092E-16	-6.069E-16	100.000
	PC14	-1.967E-16	-1.093E-15	100.000
	PC15	-2.284E-16	-1.269E-15	100.000
	PC16	-3.256E-16	-1.809E-15	100.000
	PC17	-3.474E-16	-1.930E-15	100.000
	PC18	-4.927E-16	-2.737E-15	100.000
QN	PC1	13.316	73.980	73.980
	PC2	2.656	14.756	88.736
	PC3	1.664	9.243	97.979
	PC4	0.364	2.021	100.000
	PC5	6.100E-16	3.389E-15	100.000
	PC6	5.504E-16	3.058E-15	100.000
	PC7	4.516E-16	2.509E-15	100.000
	PC8	3.818E-16	2.121E-15	100.000
	PC9	1.884E-16	1.047E-15	100.000
	PC10	7.658E-17	4.255E-16	100.000
	PC11	5.106E-17	2.836E-16	100.000
	PC12	-7.824E-17	-4.347E-16	100.000
	PC13	-9.295E-17	-5.164E-16	100.000
	PC14	-2.558E-16	-1.421E-15	100.000
	PC15	-3.312E-16	-1.840E-15	100.000
	PC16	-4.088E-16	-2.271E-15	100.000
	PC17	-4.665E-16	-2.592E-15	100.000
	PC18	-5.588E-16	-3.105E-15	100.000

Note: The PC1 to PC18 denote the 18 components of the principal component analysis, respectively.

Table S2 Loading matrix of principal components.

NO.	Quality indicators	SB		XI			QN		
		PC1	PC2	PC1	PC2	PC3	PC1	PC2	PC3
1	TSS	0.925	-0.069	0.774	-0.066	0.622	0.944	0.325	0.029
2	pH	0.983	0.108	0.787	-0.171	0.587	0.861	0.306	0.370
3	Dp	0.899	-0.434	0.963	-0.121	-0.215	0.949	-0.287	-0.105
4	Cy	0.838	-0.524	0.945	-0.115	-0.277	0.998	-0.047	-0.019
5	Pt	0.908	-0.410	0.958	-0.222	-0.184	0.749	-0.532	0.363
6	Pn	0.828	-0.530	0.921	-0.274	-0.253	0.973	-0.102	-0.171
7	Mv	0.925	-0.366	0.943	-0.273	-0.174	0.743	-0.510	-0.385
8	Pn-ac	0.936	-0.005	0.937	0.098	-0.331	0.964	-0.036	0.192
9	Mv-ac	0.858	0.511	0.037	0.907	0.252	0.965	-0.196	-0.151
10	t-Pn-co	0.792	-0.513	-0.081	0.987	0.098	0.980	-0.064	0.032
11	t-Mv-co	0.889	0.297	0.970	-0.067	-0.131	0.925	0.012	-0.377
12	TAC	0.961	0.007	0.966	-0.013	-0.253	0.969	0.131	-0.104
13	Glucose	0.866	0.072	0.776	-0.151	0.572	0.949	-0.243	0.200
14	Fructose	0.946	-0.005	0.670	-0.105	0.719	0.990	-0.022	0.119
15	esters	0.343	0.849	0.806	0.568	0.098	0.757	0.591	-0.044
16	terpenes	0.774	0.605	0.944	0.233	-0.204	-0.096	-0.308	0.947
17	high alcohols	0.461	0.810	0.749	0.572	0.251	0.797	0.551	0.173
18	aldehydes and ketones	0.686	0.652	-0.384	-0.642	0.575	0.114	0.975	0.100

Table S3 Score expressions of the 3 principal components and a model for comprehensive evaluation of grape quality

	SB	XI	QN
F1	$F1_{(SB)}=0.259X_1+0.276X_2+0.252X_3+0.235X_4+0.255X_5+0.232X_6+0.260X_7+0.263X_8+0.241X_9+0.222X_{10}+0.249X_{11}+0.270X_{12}+0.243X_{13}+0.266X_{14}+0.096X_{15}+0.217X_{16}+0.130X_{17}+0.193X_{18}$	$F1_{(XI)}=0.226X_1+0.230X_2+0.281X_3+0.276X_4+0.279X_5+0.269X_6+0.275X_7+0.273X_8+0.011X_9-0.024X_{10}+0.283X_{11}+0.282X_{12}+0.226X_{13}+0.195X_{14}+0.235X_{15}+0.275X_{16}+0.218X_{17}-0.112X_{18}$	$F1_{(QN)}=0.259X_1+0.236X_2+0.260X_3+0.274X_4+0.205X_5+0.267X_6+0.204X_7+0.264X_8+0.264X_9+0.269X_{10}+0.253X_{11}+0.266X_{12}+0.260X_{13}+0.271X_{14}+0.207X_{15}-0.026X_{16}+0.218X_{17}+0.031X_{18}$
F2	$F2_{(SB)}=-0.035X_1+0.055X_2-0.221X_3-0.267X_4-0.209X_5-0.270X_6-0.186X_7-0.002X_8+0.260X_9-0.262X_{10}+0.152X_{11}+0.004X_{12}+0.037X_{13}-0.003X_{14}+0.433X_{15}+0.309X_{16}+0.413X_{17}+0.332X_{18}$	$F2_{(XI)}=-0.037X_1-0.095X_2-0.067X_3-0.064X_4-0.124X_5-0.153X_6-0.152X_7+0.055X_8+0.505X_9+0.550X_{10}-0.037X_{11}-0.007X_{12}-0.084X_{13}-0.059X_{14}+0.317X_{15}+0.130X_{16}+0.319X_{17}-0.358X_{18}$	$F2_{(QN)}=0.199X_1+0.188X_2-0.176X_3-0.029X_4-0.327X_5-0.063X_6-0.313X_7-0.022X_8-0.120X_9-0.039X_{10}+0.007X_{11}+0.080X_{12}-0.149X_{13}-0.014X_{14}+0.363X_{15}-0.189X_{16}+0.338X_{17}+0.598X_{18}$
F3	—	$F3_{(XI)}=0.391X_1+0.369X_2-0.135X_3-0.174X_4-0.115X_5-0.159X_6-0.109X_7-0.208X_8+0.158X_9+0.061X_{10}-0.082X_{11}-0.159X_{12}+0.359X_{13}+0.451X_{14}+0.062X_{15}-0.128X_{16}+0.158X_{17}+0.361X_{18}$	$F3_{(QN)}=0.022X_1+0.287X_2-0.082X_3-0.015X_4+0.282X_5-0.133X_6-0.299X_7+0.149X_8-0.117X_9+0.025X_{10}-0.292X_{11}-0.080X_{12}+0.155X_{13}+0.092X_{14}-0.034X_{15}+0.734X_{16}+0.134X_{17}+0.077X_{18}$
F	$F_{(SB)}=0.706\ F1_{(SB)}+0.214\ F2_{(SB)}$	$F_{(XI)}=0.653\ F1_{(XI)}+0.179\ F2_{(XI)}+0.141\ F3_{(XI)}$	$F_{(QN)}=0.740\ F1_{(QN)}+0.148\ F2_{(QN)}+0.092\ F3_{(QN)}$

Note: F1 to F3 denote the score expressions of the 3 principal components, respectively. F denotes the comprehensive evaluation function of grape quality. X1-X18 denote the values of the 18 quality indicators normalised by the Z-score, respectively. — denotes that the expression is not taken into account.
